# EpCAM Aptamer-siRNA Chimera Targets and Regress Epithelial Cancer

**DOI:** 10.1371/journal.pone.0132407

**Published:** 2015-07-15

**Authors:** Nithya Subramanian, Jagat R. Kanwar, Rupinder K. Kanwar, JagadeeshBabu Sreemanthula, Jyotirmay Biswas, Vikas Khetan, Subramanian Krishnakumar

**Affiliations:** 1 Department of Nanobiotechnology, Vision Research Foundation, Kamalnayan Bajaj Institute for Research in Vision and Ophthalmology, Chennai, India; 2 Nanomedicine-Laboratory of Immunology and Molecular Biomedical Research (NLIMBR), School of Medicine (SoM), Centre for Molecular and Medical Research (C-MMR), Faculty of Health, Deakin University, Geelong, Victoria-3217, Australia; 3 L & T Ocular Pathology department, Vision Research Foundation, Kamalnayan Bajaj Institute for Research in Vision and Ophthalmology, Chennai, India; 4 Department of Ocular Oncology and Vitreoretina, Medical Research Foundation, Sankara Nethralaya, Chennai, India; Consiglio Nazionale delle Ricerche (CNR), ITALY

## Abstract

Epithelial cell adhesion molecule (EpCAM), a cancer stem cell (CSC) marker is over expressed in epithelial cancers and in retinoblastoma (RB). We fabricated an EpCAM targeting aptamer-siRNA chimera and investigated its anti-tumor property and EpCAM intracellular domain (EpICD) mediated signaling in epithelial cancer. The anti-tumor efficacy of EpCAM aptamer-siEpCAM chimera (EpApt-siEp) was evaluated by qPCR, northern and Western blotting in WERI-Rb1- RB cell line, primary RB tumor cells and in MCF7- breast cancer cell line. Anti-tumor activity of EpApt-siEp was studied *in vivo* using epithelial cancer (MCF7) mice xenograft model. The mechanism and pathways involved in the anti-tumor activity was further studied using protein arrays and qPCR. EpApt-siEp chimera was processed *in vitro* by dicer enzyme. Treatment of the WERI-Rb1 and MCF7 cells with EpApt-siEp revealed statistically significant down regulation of EpCAM expression (P<0.005) and concomitant reduction in cellular proliferation. In primary RB cells cultured from RB tumors, EpApt-siEp silenced EpCAM, significantly inhibited (P<0.01) cell proliferation and induced cytotoxicity. Knockdown of EpICD expressed in RB primary tumors led to repression of pluripotency markers, SOX2, OCT4, NANOG, and CD133. *In vivo* studies showed complete tumor growth regression without any toxicity in animals (P<0.001) and tumor tissues showed significant downregulation (P<0.05) of EpCAM, MRP1, ABCG2, stathmin, survivin and upregulation of ATM (P<0.05) leading to apoptosis by intrinsic pathway with minor alteration in cytokines. Our results revealed that EpApt-siEp potentially eradicated EpCAM positive cancer cells through CSC marker suppression and apoptosis, while sparing normal EpCAM negative adjacent cells.

## Introduction

Epithelial cell adhesion molecule (EpCAM) is a well-known cancer stem cell (CSC) marker expressed on cell surface and regarded as a tumor associated antigen[1]. EpCAM is over-expressed in epithelial tumors such as breast cancer and childhood eye cancer such as Retinoblastoma (RB)[2–4]. EpCAM is associated with increased proliferation, migration and invasion in both breast cancer and RB[5, 6].EpCAM protein is differentiated into extracellular domain (EpEx), transmembrane domain (EpTM) and intracellular domain (EpICD). It plays a vital role in oncogenic signaling by EpCAM proteolysis and EpICD translocation into the nucleus[7, 8].Proteolysis of EpCAM leads EpICD to form complex withFHL2, β-catenin and Lef1. This complex binds to theLef1 binding site of the target genes and modulates their transcription[7].EpICD is known to occupy promoter region and positively regulate SOX2, OCT4 and NANOG which contributes to self-renewal and pluripotency of cancer cells[9].

EpCAM is considered as an ideal therapeutic target to treat cancer because of the difference in its spatial distribution between normal and cancer cells. EpCAM is overexpressed in the apical surface of the tumor cells[10]and minimally in the basolateral surface of normal epithelial cells and mutations have not been described in EpCAM so far in cancer cells[11]. Several anti-EpCAM antibodies such as edrecolomab and adecatumumab were generated to target cancer and studied in clinical trials[12]. To further improve the therapeutic potential of EpCAM targeting, aptamers with greater specificity and higher affinity were sought[13].

Aptamers are synthetic oligonucleotide (RNA/ssDNA) or peptide molecules that bind to a specific target with high affinity due to their three dimensional structures[14]. They are synthesized from vast molecular libraries by a selection process called ‘Systemic evolution of ligands by exponential enrichment’ (SELEX) [15, 16]. Both RNA and ssDNA aptamers were developed against cell surface EpCAM [13, 17]. Since EpCAM RNA aptamer was shown to get internalized by endocytosis, it would be capable of delivering siRNA into the cell upon chimerization. Several aptamer–siRNA chimerization strategies were studied for targeting cancer cells. RNA aptamers against surface markers such as PSMA, EGFR, BAFF-R, integrins and DNA aptamer against nucleolin were reported for delivering siRNA in various cancer models (summarized in S1 Table).

The functionality of aptamer-siRNA chimeric constructs could be explained in three steps such as (i) binding and internalization, (ii) dicer processing and (iii) RNAi mediated silencing[18]. Previously, we have demonstrated the specific targeting of RB by using aptamer-doxorubicin (EpDT3-dox) that binds cell surface EpCAM to deliver doxorubicin[19]. Here for the first time, we have constructed EpCAM RNA aptamer–EpCAM siRNA chimera (EpApt-siEp) to achieve targeted EpCAM gene silencing in EpCAM positive cells. We also report for the first time that EpICD is over-expressed in RB, and knocking down EpCAM leads to the down-regulation of CSC markers such as SOX2, OCT4 and NANOG expression. We also performed *in vivo* studies using xenograft model in nude mice with MCF7 breast cancer cell line, as a proof of concept for solid epithelial cancers which expresses EpCAM. High anti-tumor activity was attained using our EpApt-siEp chimeric construct without toxicity. Further, we studied the mechanism involved in the cell death and inhibition of cell proliferation in the tumor xenografts by investigating the complete apoptotic and cytokines molecules using protein arrays.

## Methods

### Fabrication of chimeric construct and *In vitro* dicer cleavage assay

EpApt-siEp was constructed as described in Dassie *et*., *al*. 2009[20]. The secondary structure of the construct was studied by RNA structure v5.3[21]and Mfold software[22]. The designed constructs were commercially synthesized by Dharmacon Inc. (GE life sciences, Lafayette, CO).*In vitro* dicer assay was performed to show that EpApt-siEp chimera is being processed by dicer enzyme for the release of siRNA from the chimeric aptamer using recombinant dicer kit(Recombinant Human dicer Enzyme Kit Cat No: T510002) following the manufacturer’s instruction. The reacted products were electrophoresed and analyzed by UV transilluminator (detailed protocol provided in S1 File).

### Cell lines and primary RB cell culture and aptamer uptake study

Human RBcell line (WERI-Rb1), Breast Cancer cell line (MCF7) purchased from Riken cell bank, RIKEN BioResource Center (Ibaraki, Japan)was included in the study. Cell lines were free of mycoplasma contamination, as verified by LookOut for Mycoplasma kit (Sigma Aldrich, Bangalore). MCF7 andWERI-Rb1 cells were cultured in Dulbecco’s modification of eagle’s media (DMEM)and Rosewell park memorial institute media (RPMI-1640)mediarespectively with 10% fetal bovine serum (FBS) (Gibco, Life technologies, Bangalore, India). All cell lines were kept at 37°C in a 5% CO2 incubator.RB tumor samples from enucleated eye balls were collected as part of the therapy and utilized for research purpose anonymously. A general written consent was obtained from the parents/guardians of the patient undergoing enucleation. The study was performed in accordance to the declaration of the Helsinki, at Vision Research Foundation, after obtaining approval from Ethics Sub-Committee (Institutional Review Board) of Sankara Nethralaya eye hospital [Ethical clearance. no. 240-2010-P]. Primary RB tumor cells obtained from the enucleated eyes was dissociated by manual trituration, and cultured in RPMI media containing 20% FBS. RPMI and DMEM media were purchased from Sigma Aldrich (Sigma Aldrich, Bangalore, India). Uptake of FITC labeled EpApt-siEp by MCF7 and WERI-Rb1 cells was studied using flow cytometry and fluorescent microscopy following protocol provided in S1 File.

### *In vitro* efficacy of EpApt-siEp in cell lines

The efficacy of EpApt-siEp was evaluated *in vitro* using MCF7 and WERI-Rb1 cell lines. Briefly, 2X10^5^ cells were treated with EpApt-siEp or transfected with siEpCAM for 48h, RNA isolation followed by northern blot, qPCR for the mRNA analysis and Western blotting for the protein levels (S1 File).

### Quantitative real-time PCR, northern and Western blotting

To analyze the effect of siEpCAM and EpApt-siEp on the EpCAM expression, qPCR and northern blotting was performed using total RNA. qPCR was performed by normalizing the target gene to β-2-microgloublin(B2M)by SYBR green based method using the primer sequences as listed in S2 Table. Northern blotting was performed by electrophoresing the total RNA in formaldehyde agarose gel, transblotted using SSC buffer, probed with biotin-labeled anti-sense EpCAM RNA probe and developed using chemiluminescence method. Western blotting was performed to analyze the EpCAM protein level using standard protocol with anti-EpCAM (c-10) antibody (detailed protocol provided in S1 File).

### Immunohistochemistry

Immunohistochemistry (IHC) was performed using Novolink polymer detections system (Leica biosystems, Bangalore, India) following the instructions given by the manufacturer using de-paraffinized human RB tissue sections. Briefly, antigen retrieval was done using pressure cooker method, then tissue peroxidase blocking and primary blocking was performed followed by incubation with the anti-EpICD antibody (Imgenex, India). Polymer based detection using DAB chromogen was done, dried slides were mounted and scored for the expression levels (detailed protocol provided in S1 File).

### MTT cell proliferation assay

Equal numbers of MCF7 and WERI-Rb1 cells (10,000 per well) were seeded respectively in a 96 well plate. After 24h, the cells were treated with 400nM aptamer-siRNA chimera. The cells were also transfected using lipofectamine 2000 (Invitrogen lifescience, Bangalore, India) with 200nM EpCAM siRNA (Qiagen, Germany). The treated cells were incubated for 48hat 37°C in 5% CO2 incubator. After 48h, MTT (Sigma Aldrich, Bangalore, India) in fresh media was added to the cells and incubated for 4h. The crystals formed were dissolved in DMSO and absorbance was read at 570nm using spectramax spectrophotometer.

### *In vivo* anti-tumor efficacy of EpApt-siEp in epithelial cancer xenograft model

To study the *in vivo* efficacy of EpApt-siEp, MCF7, epithelial cancer model was chosen since the *in vitro* efficacy was better than WERI-Rb1 cell line. This study was performed in the premises of Syngene International Pvt. Ltd., commercially. All animals were handled in a manner to minimize or eliminate pain and suffering by using isoflurane based anesthetization. Animal care was in compliance with the recommendations of Committee for the Purpose of Control and Supervision of Experiments on Animals (CPCSEA), Government of India and Association for Assessment and Accreditation of Laboratory Animal Care International (AAALAC). The ‘Form B’ for carrying out animal experimentation was reviewed and approved by the Syngene International Pvt. Ltd., Institutional Animal Ethics Committee (IAEC Protocol Approval No: SYNGENE/IAEC/430/10-2013). Animals were maintained in controlled and aseptic condition and provided with corncobs, RO water autoclaved ad libitum and with light/dark cycle of 12h each. MCF7cells were suspended at a concentration of 5X10^6^cells in 200μl of serum free media containing 50% of Matrigel and injected subcutaneously in the back of athymic nude-Foxn1^nu^ovariectomized female mice of 7–8 weeks old implanted with 17β-estradiol pellets. Once the tumors became palpable, animals were randomly grouped based on the tumor volume (TV≈80mm^3^) and dosing was initiated. The treatment schedule followed is given in S3 Table. The body weights and tumor volume were measured once every three days and % change in body weight was calculated. During sacrifice, blood was collected under isoflurane anesthesia from all groups for clinical assessment of liver function (SGOT, SGPT) & kidney function (BUN, Urea), peripheral blood smear for differential leucocyte count. Tumor tissues and organs were excised and analyzed histo-pathologically by haemotoxylin and eosin staining.

### Protein array for apoptotic markers and cytokines

Proteome profiling was performed to study the mechanism of EpApt-siEp construct mediated anti-tumor activity and to study its effect on apoptosis onset and inflammatory response. The protein arrays–Human apoptosis array, catalog# ARY009 and Mouse cytokine array panel A catalog# ARY006 (R&D Systems, Abingdon, UK) were performed following the manufacturer’s instruction. The xenograft mouse (vehicle control—treated with sterile water for injection and EpApt-siEp treated) tissues protein lysates and serum were prepared by normalizing their protein concentration and used in the array. The arrays were performed at an identical condition and developed simultaneously for both the control and treatment groups using chemidoc XRS^+^ instrument (BioRad) using same exposure. Background signal normalization was performed and integrated pixel density was measured using imageJ software with the microarray profile plugin. The differences between the duplicate spots were used for calculating the standard deviation and expressed as error bar in the histogram plots.

### Statistical analysis

Statistical analysis for the *in vitro* analysis of aptamer chimera on cell lines was performed with unpaired *t*-test. The experiments were carried out in triplicates and repeated thrice. For the evaluation of the statistical significance of tumor inhibition, unpaired *t*-test was performed using Graph Pad Prism v5. The tumor inhibition studies were performed in xenograft mice with n = 8. The p values less than 0.05 indicates statistically significant differences between groups. P value in the range (0.01–0.05)is indicated with “*”, values in the range(0.01–0.001) with “**” and less than 0.001 is indicated with “#”.

## Results

### Chimerization of EpApt with siEpCAM; *in vitro* dicer mediated processing and cellular uptake

**Fig 1:**
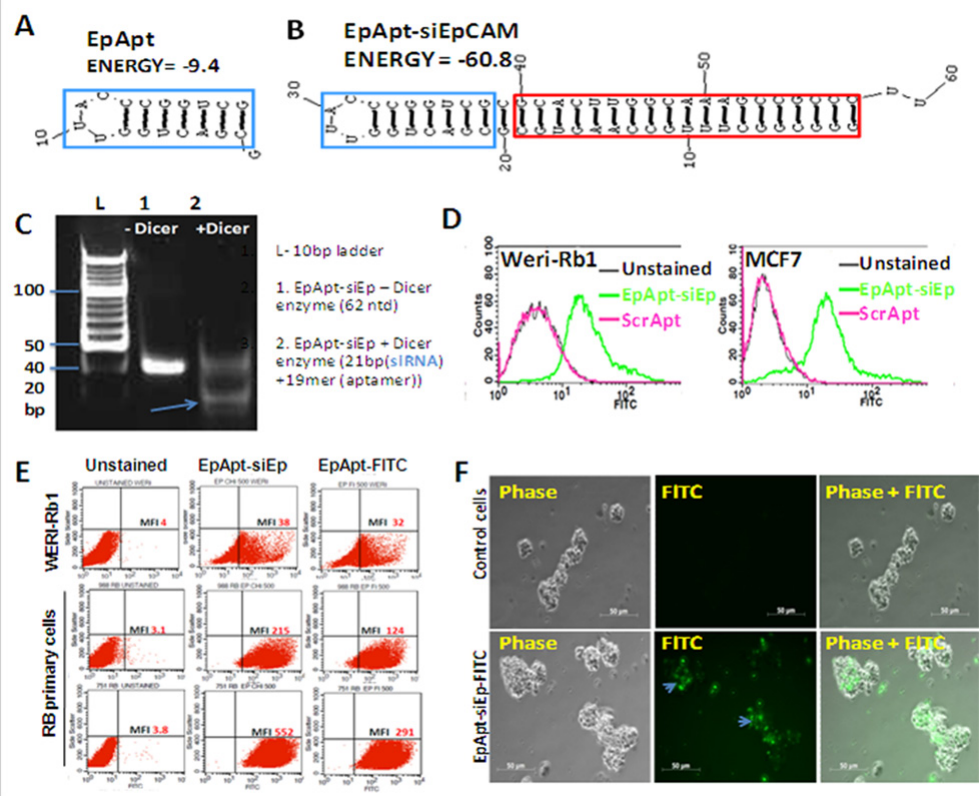
EpApt-siEp fabrication, *in vitro* processing by dicer enzyme, cell surface binding and internalization. **A**. EpCAM aptamer secondary structure prediction from Mfold online. **B**. EpCAM aptamer siRNA chimeric construct carrying the siRNA targeting EpCAM (EpApt-siEp) is folded using Mfold online and the aptamer is indicated in blue box and the siRNA inside red box. **C**. EpCAM aptamer siRNA chimeric construct was incubated with the recombinant dicer enzyme at 37˚C for 18h. The reactions were performed without dicer as control reaction. Polyacrylamide gel electrophoresis of the reactions with and without dicer enzyme were run on 15% gel and stained with EtBr. The processed 21bp siRNA and unprocessed construct were observed. **D**. EpCAM aptamer siRNA chimeric construct was added to WERI-Rb1 and MCF7 cells in binding buffer and analyzed by flow cytometry. The overlay graph shows the uptake of the chimeric aptamer. **E**. Scatter plot showing the uptake of EpApt-siEp by the RB cell line, WERI-Rb1 and RB primary tumor cells. **F**. EpCAM aptamer siRNA chimeric construct was added to primary RB cells in media without serum for 2hr at 37˚C followed by washing with 1X PBS. Microscopic images were taken at 20X objective under phase and FITC channels of control cells alone and cells with EpApt-siEp. Data represents mean ± SD. Experiments were repeated 3 times independently with similar results. **P value of 0.01–0.001; *P value of 0.05–0.01.

EpCAM aptamer siRNA chimera was fabricated following the previously optimized structures of PSMA aptamer siRNA chimeric construct [20]. In the previous study, stem and loop aptamer chimera with strand swap exhibited better silencing compared to the other chimeric forms. Hence in the current study, we fabricated aptamer chimera by extending the aptamer sequence with siRNA sequence at its 5’end and 3’end. The fabricated EpApt-siRNA carried siRNA targeting EpCAM. The stem and loop structure was designed manually by incorporating siRNA sequence targeting EpCAM. Additionally the aptamers-siRNA carried nuclease resistant modification (2’F) in the pyrimidines. The 5’end of the aptamer was end labeled with FITC to monitor the aptamer binding and uptake by cells and 3’end of the aptamer harbored two uridine overhangs that aids in the recognition and loading of dicer enzyme[23]. The EpApt and constructed aptamer chimeric structures were predicted using RNA structure v5.3 and Mfold and presented in Fig 1A and 1B. The EpApt aids in binding to EpCAM, internalization and release into cell cytoplasm. The EpApt-siEp under the influence of dicer, generates21bp siRNA that loads into the RISC complex, orchestrates inhibition of translation by EpCAM mRNA cleavage.

For the functionality of synthesized EpApt-siEp, dicer recognition is necessary. This was tested by performing *in vitro* dicer cleavage assay. The EpApt-siEp construct was incubated with recombinant human dicer for 18h and subsequently electrophoresed on agarose gel. The results revealed 21bp and 19merproducts corresponding to siRNA and aptamers respectively (S1 Fig). This was further confirmed by running a non-denaturing PAGE, cleaved fragments of ~20–22bp length were obtained (Fig 1C). We further sought to examine the stability of the construct under physiological mimicking conditions. The construct resulted in minimal degradation (<10% degradation) till 72h in media without and with 10% FBS respectively. The construct in 100% FBS was stable till 96h, although, a slight degradation was evident at 48h duration. Thus the constructs could be stable under physiological mimic conditions till 72h (S1A Fig).

The cellular uptake studies of EpApt-siEp are necessary to substantiate the internalization of aptamer through receptor mediated endocytosis. The EpCAM aptamer (EpDT3/EpApt) has already been elucidated for the receptor mediated endocytosis[13].Earlier studies and current study used EpCAM scramble aptamer (ScrApt), with 2’OMe modification in the EpApt backbone hinders the binding to EpCAM[13, 19]. Similar to the EpApt-siEp, ScrApt chimera (ScrApt-siEp) was constructed. The ScrApt-siEp upon chimerization resulted in non-specific binding to MCF7 cells (S1B Fig) and not investigated further. We found higher cellular uptake of EpApt-siEp construct in MCF7 than WERI-Rb1cells (Fig 1D). The primary RB cells and the WERI-Rb1 cell line showed uptake of chimera. The primary RB cells were found to exhibit higher binding efficiency than cell lines, due to higher levels of EpCAM expression in the tumors (Fig 1E). From the microscopic studies 75% of primary RB cells showed uptake of the EpApt-siEp (Fig 1F).

### EpApt-siEp silences EpCAM specifically in cell lines and primary RB tumor cells

**Fig 2:**
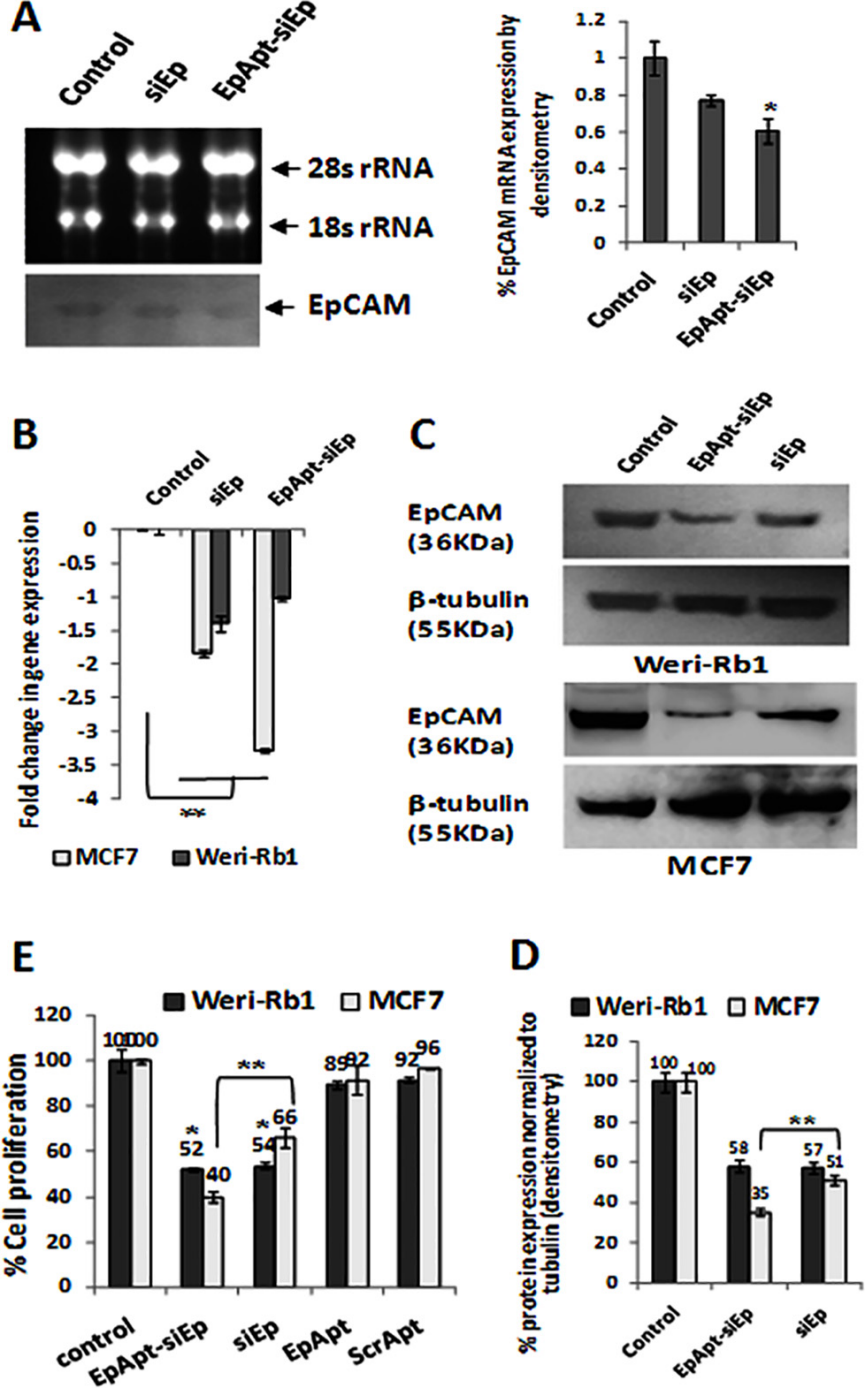
EpCAM knockdown using EpApt-siEp construct in WERI-Rb1 and MCF7 inhibits cell proliferation. **A.** The EpCAM mRNA levels were detected from the total RNA of control, siEp and EpApt-siEp treated MCF7 cells by northern blotting. The total RNA was electrophoresed in formaldehyde agarose gel electrophoresis, blotted and developed by chemiluminescence based method. EpCAM targeting siRNA was used for synthesizing probe. On its right, the densitometry analysis of the bands were performed using imageJ software and plotted as graph with % mRNA expression against the 28s rRNA. **B**. The EpCAM mRNA levels were quantified by SYBR green based qPCR from the cDNA of control, siEp and EpApt-siEp treated WERI-Rb1 and MCF7 cells. **C**. Western blotting was performed on the siEp transfected and EpApt-siEp treated WERI-Rb1 and MCF7 cells for the EpCAM and b-tubulin. EpCAM targeting siRNA was used for synthesizing probe. **D**. The densitometry analysis of the western blotting bands was performed using imageJ software and plotted as graph with % protein (EpCAM) expression normalized to β-tubulin. **E**. The percentage cell proliferation was quantified by performing MTT assay on the control, siEp transfected, EpApt-siEp, EpApt and ScrApt treated WERI-Rb1 and MCF7 cells. The graph shows the % cell proliferation normalized to control cells. Data represents mean ± SD. Experiments were repeated 3 times independently with similar results. **P value of 0.01–0.001; *P value of 0.05–0.01.

The target specific delivery, siRNA generation and silencing capability were studied using WERI-Rb1 and MCF7 cell lines. Since the expression level of EpCAM is higher in MCF7 cells than WERI-Rb1[24], the chimeric construct was first evaluated for the silencing of EpCAM in MCF7 cells. The northern blotting results showed silencing of EpCAM about 40% in EpApt-siEp treated cells and around 25% in siEp transfected cells normalized to 28s rRNA (Fig 2A and panel right to it). A quantitative analysis of the mRNA expression by qPCR showed better inhibition of EpCAM expression, -1.8 and -3.4 fold downregulation (73% and 90% inhibition of mRNA expression) in MCF7 cell line (P<0.01) and -1.4 and -1.0 fold downregulation (63% and 51% inhibition of mRNA levels) in WERI-Rb1cell line (P<0.05) (Fig 2B). The EpCAM downregulation was also observed at the protein levels (Fig 2C), WERI-Rb1 cells exhibited 47% and 43% and MCF7 exhibited 65% and 49% of downregulation of EpCAM protein (P<0.05) (Fig 2D). The unprocessed northern and Western blots are shown in S2 File.

The effect of chimeric construct was tested in RB primary tumor cells for silencing. The functional/metabolic activityof primary cells wasevaluated bytransfecting pGFP plasmid. 24h post transfection majority of the cells showed very high expression (S2A Fig). This confirmed the metabolically active state of the primary cells, we then sought to analyze the effect of EpApt-siEp and siEp on these cells. The primary tumor cells showed inhibition of EpCAM expression by -0.5 fold and -2.4 fold in siRNA and chimeric construct treated cells respectively (S2B Fig). The cellular cytotoxicity as measured by LDH assay showed 37% and 35% increase in the LDH activity upon silencing of EpCAM using siRNA and EpApt-siEp. The EpApt-siEp construct significantly (P<0.01) downregulated EpCAM mRNA levels and caused cytotoxicity in primary RB tumor cells (S2C Fig). The cell proliferation as a read out of metabolic activity was measured by MTT assay. TheWERI-Rb1 and MCF7 cells showed significant cell proliferation inhibition with EpApt-siEp, while EpApt or ScrApt alone did not show any effect in cell proliferation (Fig 2E).

### EpICD in primary RB tumors: regulation of cancer stem cell markers

**Fig 3:**
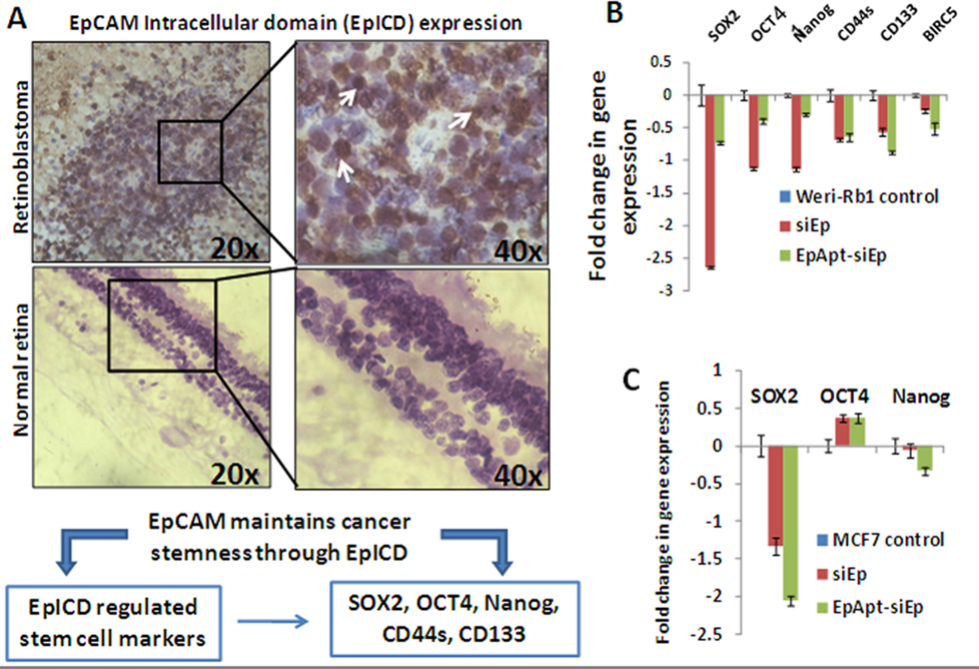
Expression of EpCAM Intracellular domain (EpICD) in RB and the effect of EpCAM knockdown on cancer stem cell markers. **A**. Immunohistochemistry of the normal retina section showing no evident EpICD in the nucleus, RB tissue sections showing intense staining of nucleus. The expression of EpICD was majorly observed in nucleus of the tumor cells as shown by white arrows. **B.** The fold change in mRNA levels of SOX2, OCT4, Nanog, CD44S, CD133 and Survivin (BIRC5) were quantified by SYBR green based qPCR from the siEp and EpApt-siEp treated WERI-Rb1 cells and normalized to β-2-microglobulin as housekeeping gene. **C**. The fold change in mRNA levels of SOX2, OCT4 and Nanog were quantified by SYBR green based qPCR from the siEp and EpApt-siEp treated MCF7 cells and normalized to β-2-microglobulin as housekeeping gene.Data represents mean ± SD. Experiments were repeated 3 times independently with similar results.**P value of 0.01–0.001; *P value of 0.05–0.01.

EpCAM has been shown to be overexpressed in cancer initiating cells or cancer progenitor/stem cells (CPC/CSCs) [25–27]. The mechanism behind this property was regulated by the intramembrane proteolysis of EpCAM leading to the EpICD release and displacing to nucleus [7].We reported the presence of EpCAM earlier in 2004 and in the current study we sought to study the expression of EpICD in primary RB tumors[28]. The IHC results showed intense nuclear staining and the intensity of the nuclear staining varied among the tumors studied. The tumors showed 40–60% of cells positive and some cases showed 70–80% of cells positive for nuclear staining. The normal retina studied did not reveal any evident nuclear staining (Fig 3A). The intensity and the percentage distribution of the EpICD positive cells in the tumor are presented in Table 1.

**Table 1:** Expression of EpICD in RB primary tumor by IHC.

Tumor details	Intensity	Percent Positivity
314/10	++++	90
435/03	+++	70
1002/03	+++	60
423/03	++	50
515/08	+++	65
935/10	+	20–30
403/08	++	40
679/10	++	60
544/10	+++	70–80
153/10	+++	70
520/11	+++	80

*+ = 20–30 number of cells have positive staining (%)*.

*++ = 31–60 number of cells have positive staining (%)*.

*+++ or more = 61–80 number of cells have positive staining (%)*.

*++++ = 81 or higher number of cells have positive staining (%)*.

The released EpICD forms nuclear protein complex by interacting with the FHL2, β-catenin and Lef1 mediates gene transcriptions and aids in cell proliferation. The regulation of EpICD on the expression of pluripotency markers were reported earlier[9]and we were interested to study the modulation of EpCAM behind the expression of OCT4, SOX2, NANOG, CD133, CD44s. We additionally studied the expression of survivin levels upon silencing of EpCAM in RB cell line, WERI-Rb1. EpCAM silencing using the siRNA transfection or EpApt-siEp showed higher downregulation of SOX2, OCT4 and NANOG in siRNA transfected cells compared to the EpApt-siEp, whereas the CD133, CD44s and survivin levels were more downregulated in EpApt-siEp transfected cells (Fig 3B). We additionally studied the expression of SOX2, OCT4 and NANOG in MCF7 cells and found downregulation of SOX2 and NANOG but not OCT4 upon silencing EpCAM using siRNA or EpApt-siEp construct (Fig 3C). Thus we were able to elucidate the regulation of stem cell markers by EpCAM through EpICD.

### EpApt-siEp regress breast cancer: *In vivo* xenograft study

**Fig 4:**
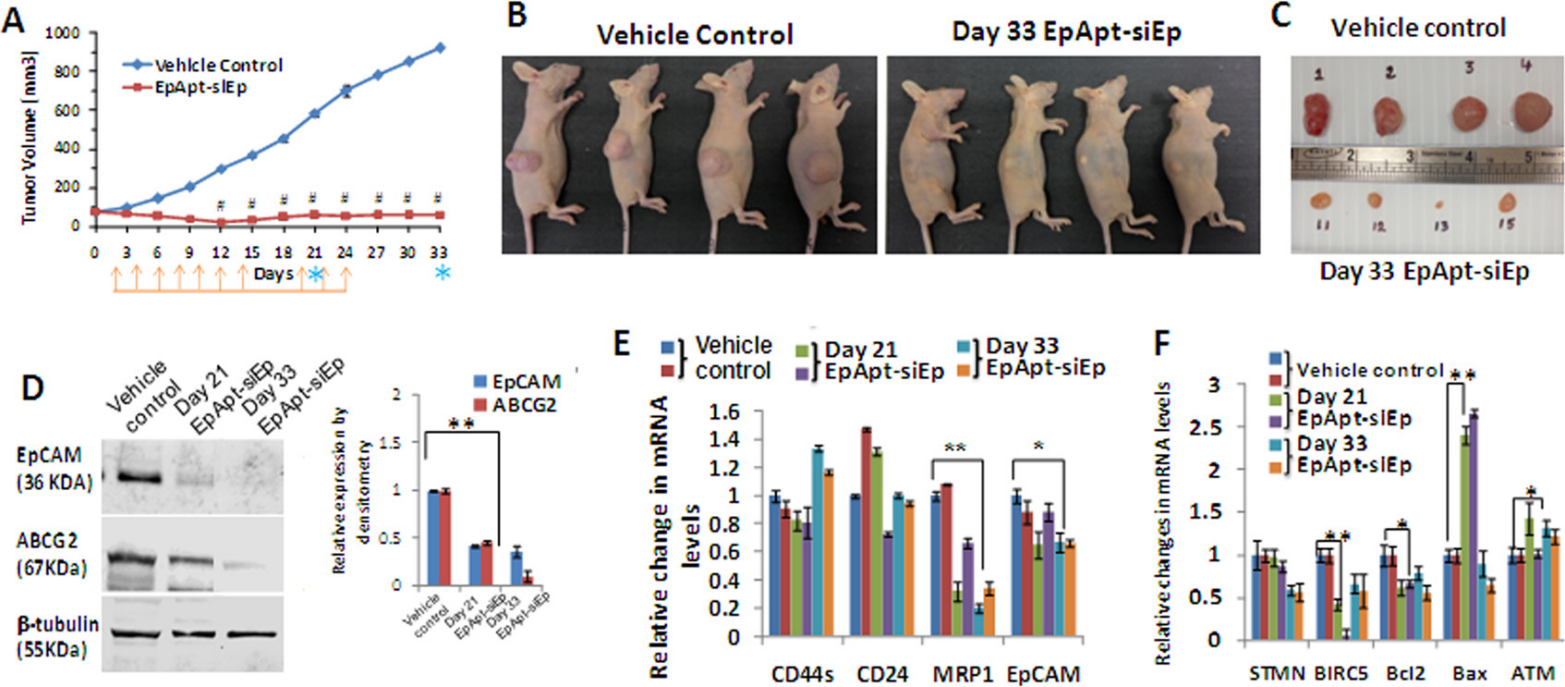
Tumor growth kinetics, changes in gene and protein expression in MCF7 Xenograft treated with EpApt-siEp. Tumor growth kinetics of MCF7 Xenograft treated with EpApt-siEp. Female nude mice (Hsd: Athymic Nude-Foxn1^nu^, bilaterally ovariectomized) housed in Individually Ventilated Cages (IVCs) were used for the present investigation. The tumorigenicity of the MCF7 cells in mice is estrogen-dependent. Twenty hours prior to MCF-7 cell injection, animals were implanted with 17β-estradiol pellets (0.36mg/pellet; 60-day release; Innovative Research of America, Sarasota, FL) into dorsal shoulder blade region of mice using trochar. MCF-7 tumor cells (5 x10^6^ cells/animal) were injected subcutaneously in the flanks of the animals. After 7–10 days post injection of cells, animals were randomized based on tumor volume (TV≈80mm3) and dosing was initiated. Graph showing the (**A**) Tumor volume of the Vehicle control group injected with PBS subcutaneously near the tumor site, EpApt-siEp subcutaneously injected near the tumor site on alternate days. The orange arrows indicate the EpApt-siEp injections given and the blue asterisk indicates the day of sacrifice. On Day 21 and 33, due to experimental and ethical reasons animals from both the groups were sacrificed 50% at each time. Photographs of the representative mice **(B)** and excised tumors **(C)** of vehicle control and treated groups. **D**. Changes in protein expression by Western blotting of proteins extraction from the representative tissue of the control mice or mice treated with EpApt-siEp (0.6nmol) and terminated at 21 and 33days respectively (on its right, graph representing the relative expression calculated by imageJ software. **E**. Graph showing the changes in MCF7 xenograft tumor tissue EpCAM, CD44s, CD24 and MRP1 mRNA levels post treatment with EpApt-siEp construct. The vehicle control was used for normalizing the fold expression and the β-2 microglobulin was used as internal control. **F**. Graph showing the changes in STMN, BIRC5, Bcl2, Bax and ATM mRNA levels post treatment with EpApt-siEp aptamer construct normalization was done with both vehicle control / no treatment group. Data represents mean ± SE for *in vivo* experiment (n = 8) and mean ±SD for other experiments. Experiments were performed in triplicates and significance was calculated by t-test. # P<0.001; **P value of 0.01–0.001; *P value of 0.05–0.01.

The anti-tumor effect of the EpApt-siEp was studied using the breast cancer *in vivo* model. MCF7 cells were injected in bilaterally ovariectomized nude mice supplemented with external estrogen. The dosing of EpApt-siEp was performed on alternate days from day0 to day14 and on day20, animals were dosed, 24h later n = 4 were sacrificed from each group. The tumor growth kinetics showed significant reduction (P<0.01) in tumor volume, vehicle control showed 584mm^3^, while the treated animal showed 52mm^3^ mean tumor volume. The rest of n = 4 in vehicle control group and EpApt-siEp group were dosed on day22 and day24, further studied till day33. The mean tumor volumes on day33, for vehicle control group and EpApt-siEp were 922mm^3^ and 64mm^3^ respectively. The tumor growth profile for both the groups during this period is shown in Fig 4A. The % tumor growth inhibition (TGI) for EpApt-siEp group at the tested dose level was found to be 102% (Day33, ^#^indicates p<0.001). On the day33, the mice (Fig 4B) were euthanized and the tumors were excised (Fig 4C) followed by analysis of the expression of EpCAM and cancer stem cell markers, apoptotic makers and drug resistant proteins were carried out.

The effect of EpApt-siEp construct on the expression of EpCAM and other CSC markers were studied between vehicle control and treated group (n = 2) tumor sections collected from day21 and day33 respectively. To check the effect of EpCAM silencing induced by EpApt-siEp construct, the expression of EpCAM was analyzed by Western blot at protein level, additionally ABCG2 protein was included. The densitometry analysis showed 55% and 60% of EpCAM protein downregulation in day21and day33 group, while ABCG2 protein showed 60% and 85% of downregulation in day21 and day33 respectively in EpApt-siEp treated samples compared to vehicle control (Fig 4D). The gene expression analysis using qPCR showed downregulation of EpCAM and MRP1 significantly on the day21 and day33 treated tumors. The levels of CD44s were high on day33 group, while day21 group showed lesser expression than vehicle control (Fig 4E). Additionally, levels of Bax, Bcl2, stathmin (STMN1), survivin (BIRC5) and ATM were studied. The expression levels of Bcl2 was found to be downregulated, while the Bax was upregulated (approximately 2.5 fold) in the day21 group, with no significant difference in the expression on day33 group. The level of ATM gene expression was significantly upregulated in 50% of day21group and 100% of day33 group, which signifies the mechanism behind the tumor suppression. Similarly the levels of stathmin and survivin were downregulated in the day21 and day33 group, the stathmin levels were significantly downregulated on day33 group, while survivin was downregulated at higher levels in day21 than the day33 group (Fig 4F).

**Fig 5:**
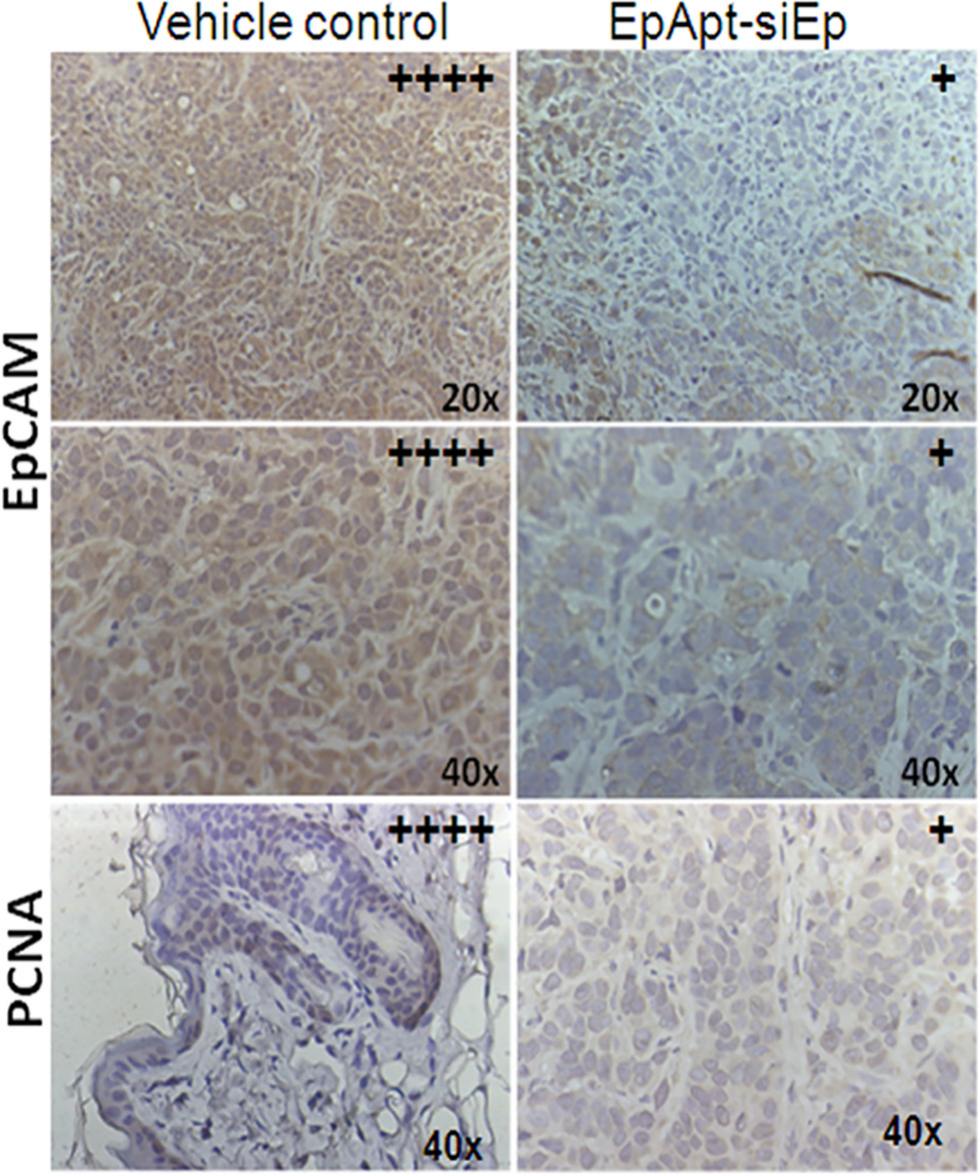
Immunohistochemical staining of xenograft tissues. Immunohistochemistry of the tumor tissues excised from the vehicle control mice and the mice treated with EpApt-siEp by intraperitoneal mode of injection. The levels of EpCAM and PCNA were studied and images are taken under 20x and 40x objective for EpCAM, 40x for PCNA. The intensities of expression of the antigens were represented with “+” on the right hand corner of the respective section.

IHC studies confirmed the downregulation of the EpCAM and proliferating cell nuclear antigen (PCNA) in the EpApt-siEp treated tissues (Fig 5). Images taken under higher objective clearly shows cytoplasmic and nuclear expression of EpCAM in control group, while faint cytoplasmic staining is observed in treated tissues. Intensely stained apoptotic bodies were observed in the treated tissues showing the possible mechanism by which the tumor regression occurred. Thus we were able to elucidate the EpCAM down regulation mediated anti-tumor effect of EpApt-siEp. The mean body weight changes from the day 0 to day 33 of vehicle control and treated mice showed no significant changes between the groups (S3A Fig). The results of DLC following treatment (EpApt-siEp) showed no significant changes compared to vehicle control group. This indicates that there was no possible evidence for the depression of bone marrow. Also there were no significant changes in the Liver function (SGOT, SGPT) & kidney function (BUN, Creatinine) parameters in EpApt-siEp treated group compared to the vehicle control group (S3B & S3C Fig). Histological observation of tumor sections, lungs, liver, spleen, kidney & heart revealed no significant difference between the study groups (S3D Fig). Thus there is no possible evidence of treatment related organ toxicity (Liver/Kidney).

### Immunomodulatory and apoptotic effect of the EpApt-siEp chimera

**Fig 6:**
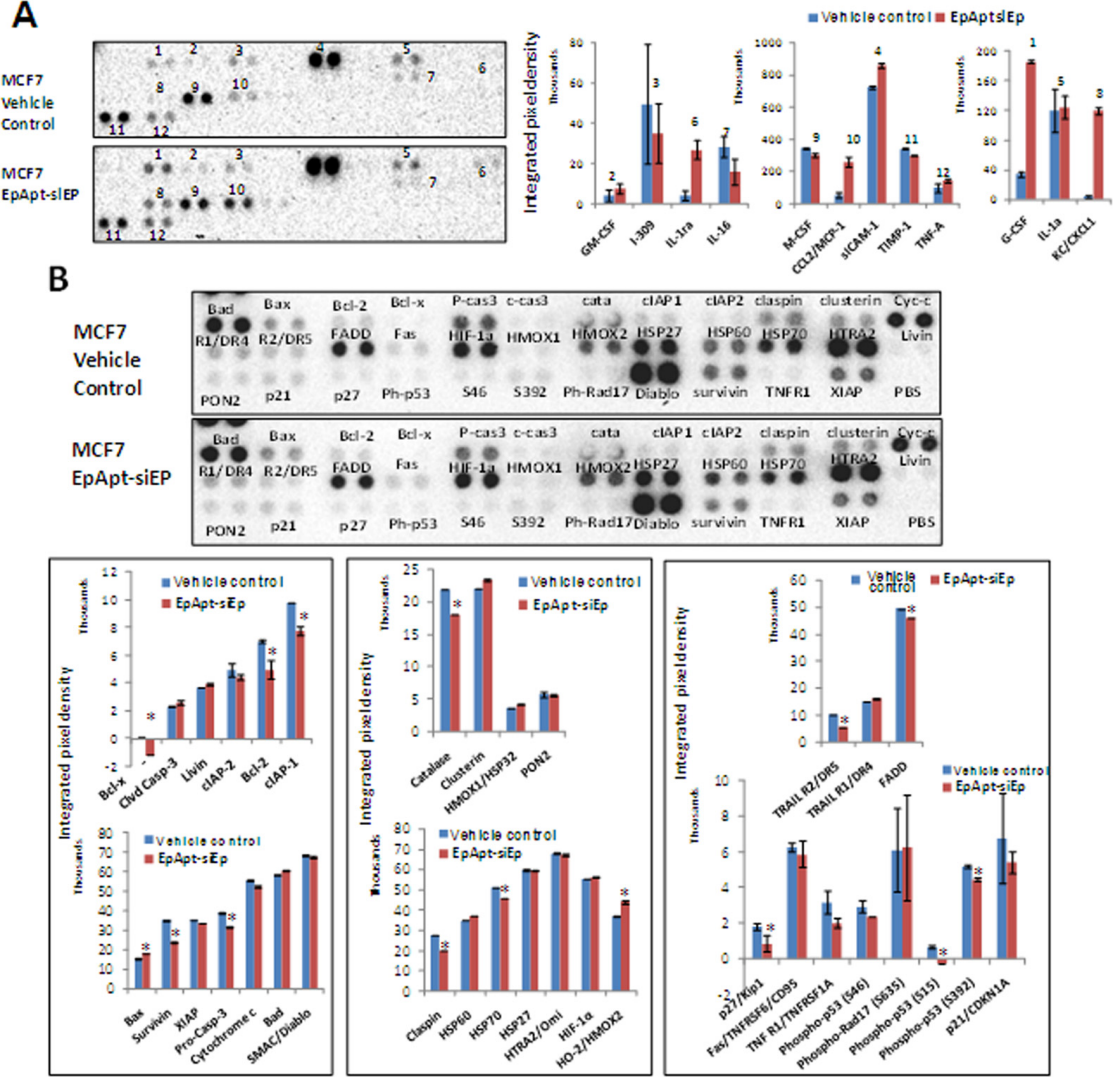
Protein array for apoptotic markers, cytokine secretion and the IHC analysis on EpApt-siEp treated xenograft samples. **A**. Mouse cytokine array performed on the vehicle control and EpApt-siEp treated mice serum collected on day 33 before sacrifice. The upper panel shows the MCF7 xenograft vehicle control serum and lower to it is the serum from mice treated with EpApt-siEp upto 24days and studied upto 33days. Graphs below shows the mean integrated pixel density of the each protein spot from the blot detected using chemiluminescence imaging and quantified using imageJ software using microarray profile plugin. **B.** The levels of apoptotic markers between the vehicle control mice and the EpApt-siEp treated mice upto 24days and studied upto 33days, was analyzed using 'human apoptotic array'. The labels next to the spot represent the protein and the integrated pixel density was quantified using imageJ software and plotted as graph (i, ii & iii). ‘i’ panel represents the targets that belongs to intrinsic apoptotic pathway, ‘ii’ panel represents extrinsic apoptotic pathway and ‘iii’ panel represents other key regulators of apoptosis. The error bar represents the standard deviation and the ** indicates P value of 0.01–0.001; * indicates P value of 0.05–0.01.

The chimeric RNAs or long RNAs are known to elicit the immunity; hence we particularly wanted to study the effect of EpApt-siEp in the cytokines expression of the mouse serum of vehicle control and EpApt-siEp treated day33 groups. The cytokine array showed significant (*, P<0.05; **, P<0.001) increase in the expression of IL1RA, CCL2, G-CSF, CXCL1and sICAM-1, while decrease in the levels of IL-16, M-CSF and TIMP-1 in the treated group compared to controls (Fig 6A). The apoptosis array revealed difference in the expression of proteins of extrinsic, intrinsic and other regulators of apoptosis (Fig 6B). In the intrinsic pathway, Bcl-xl, Bcl2, cIP-1, survivin was significantly downregulated with significant increase in the Bax expression. There was upregulation of cleaved Caspase-3, Bad and downregulation of pro-caspase-3 and cIAP-2 (Fig 6B i). The extrinsic pathway regulators were not significantly altered though FADD and Trail2 showed decrease in expression. There were only minor changes in the extrinsic pathway regulators, but the levels of HSP60 was elevated, while HSP70, claspin and catalase were downregulated, hallmark proteins that inhibits the apoptosis (Fig 6B ii, iii). Thus the EpApt-siEp was found to mediate apoptotic cell death through intrinsic pathway.

## Discussion

Aptamers are class of next generation therapeutics[29], and RNA aptamers are more preferred as they are easy for chimerization and targeting the functional RNA molecules such as siRNAs, shRNAs, miRNAs and ribozymes[30–32].Nevertheless, DNA aptamers were also equally exploited for cancer targeting[33]. The backbone modification of aptamers to yield increased stability *in vivo* conditions and 2’Fluoro(2’F), 2’OMethyl(2’OMe) of bases and 3’ inverted thymidine(idT) are proved to be essential for nuclease resistance. The S1 Table contains the list of aptamer and siRNA chimerization based on oligonucleotides approach reported until now. Studies on liposome encapsulated siRNA, using protein tag, polyethylene glycol (PEG) and polyethylenimine[24, 34]based chimeric nanoformulation for targeted delivery are also reported [30, 35, 36].We hypothesize to target CSCs by delivering siRNA using the EpCAM aptamer in the present study. In spite of the availability of CD133[37]and CD44 aptamers[38], EpCAM is of interest due to its role in the maintenance of pluripotency, undifferentiated state of the stem cells[26] and over-expression in cancer cells [1, 39].

EpCAM is over-expressed in all epithelial cancers and in RB. Earlier, we used EpApt for the delivery of doxorubicin to RB cells sparing non-malignant cells[19] and then utilized bio-orthogonal chemistry based labeled EpCAM DNA aptamer(EpD) for imaging of cancer cells [40].We showed chimeric EpCAM and nucleolin aptamer targeted super paramagnetic iron oxide nanoparticles(SPION) saturated lactoferrin, with promising anti-tumor property *in vitro* and *in vivo*[41]. The current study adapted the optimized structure for EpCAM aptamer as reported by Dassie *et al*. 2009 to successfully fabricate and elucidate target specific silencing of EpCAM using EpApt-siEp chimeric construct. This method of chimerization, avoids the overhead of annealing separate strands to generate chimeric constructs as reported earlier [42].

The EpApt-siEp chimeric construct carrying the nuclease resistant modification increased the stability and prevented the degradation of the aptamer under physiological condition upto 72h. The 2’F modification provides stability without harming the structure, or tertiary folding. This modification prevents the exposed 2’OH of the ribose from ribonucleases and exonucleases in the serum and body fluids. Thus the oligonucleotides modified with 2’F modification carry better stability and prevented from degradation. The chimeric construct was able to silence the EpCAM in both the WERI-Rb1 cells and MCF7 cell lines. Also, the metabolically active primary RB tumor cells showed high transfection efficiency in peripheral cells. This could be due to the spheroid like nature of the primary cell cluster and the cellular arrangement which prevents the transfection complex to penetrate in the inner core. The silencing mediated by the EpApt-siEp construct was higher than the siEp transfection due to the penetrating characteristics of the EpApt-siEp construct in 3D cellular arrangement. This could be the potential mechanism adopted in the *in vivo* xenograft system for the tumor penetration and cellular internalization. The functional activity of the EpApt-siEp construct in primary tumor cells, WERI-Rb1 cells and MCF7 cell lines could be mediated by the cell proliferation inhibition induced cytotoxic effect. Further studies are needed to elucidate the possible mechanism behind the cytotoxic effects.

Until 2009, the role of EpCAM in cell proliferation was not elucidated and later it was found to be regulated through the intramembrane proteolysis and release of EpICD, that translocate to nucleus for mediating transcription of gene that promotes cell proliferation[7].Hence, we anticipated EpCAM targeting with chimeric construct will have higher anti-tumor activity, as the EpCAM expressing cells can only uptake the construct and result in cell proliferation inhibition mediated by EpCAM silencing. Thus our construct undergoes double selection and specifically acts on EpCAM expressing cells leading to less off-target effect.

The role of EpICD in the stem cell signaling is elucidated previously [9, 26]. EpICD expression in cancer cells may help to gain resistant phenotype, that further lead to resistance to chemotherapy. With this background, our results show that upon silencing of EpCAM in RB, the pluripotency markers (OCT4, SOX2 and NANOG) and cell surface markers (CD133 andCD44) gets downregulated. The levels of survivin were significantly downregulated in chimeric construct treated cells. In case of breast cancer cell line (MCF7), there was no significant change in OCT4 and NANOG expression upon silencing EpCAM, while SOX2 was downregulated. This phenomenon observed could be due to the EGFR presence in the MCF7 cells and earlier studies showed the effect of EGFR signaling mediated alteration in SOX2 expression with mild or no changes in OCT4 and NANOG. The downregulation of SOX2 alone was enough to bring down the self-renewable capacity of cancer cells with stem like characteristics [43].

The *in vivo* assessment of the anti-proliferative and anti-tumor property of the chimeric construct dosed in alternate days upto 14days followed by next cycle of dosing from day 20, 22 and 24 monitored till 33days resulted in tumor regression. The chimera revealed high anti-tumor activity without toxicity in animals. The biochemical tests for liver, kidney function and blood cell counts revealed the potentiality to use the chimera safely *in vivo*. The treated groups after day24 dosing were monitored till day33 for any regrowth of tumors. Interestingly, tumor was not developed in mice treated with chimeric construct. The molecular analysis of the excised tumors treated with EpApt-siEp chimeric construct showed downregulation of EpCAM at mRNA and protein level. We further analyzed the excised tumor for series of CSC markers (CD44, CD24 and ABCG2). The gene contributing majorly to the oncogenesis of breast cancer (survivin, stathmin and Bcl2) were significantly downregulated upon treating with chimeric construct. The downregulation of stathmin, survivin would have synergistically functioned for the anti-proliferative effect of cells.

Remarkable increase in the expression of Bax was observed on day21 group, but in day33 group the expression lowered down. Increase in Bax expression at day21 shows the EpApt-siEp mediated apoptotic onset, while the expression decreased at day33 attributing to inactive state of treated tumor cells. The chimeric construct induced Bax expression and decreased Bcl2 expression. The decrease in motility related protein1 (MRP1) also reveals the mode of the anti-tumor property exhibited by the chimeric construct. Also 50% of the treated population showed significant increase in expression of ATM levels which is generally downregulated in the breast cancer. The levels of ATM and the poor prognosis of the disease have been reported previously [44].Hence the increase in ATM expression upon chimeric construct treatment is an interesting finding for molecular mechanism in breast cancer. There is positive correlation between the EpCAM and mir-17-92 cluster over-expression in cancers [45]and mir-18a targets ATM, thereby downregulating its expression to aid tumorigenesis [46].

**Fig 7:**
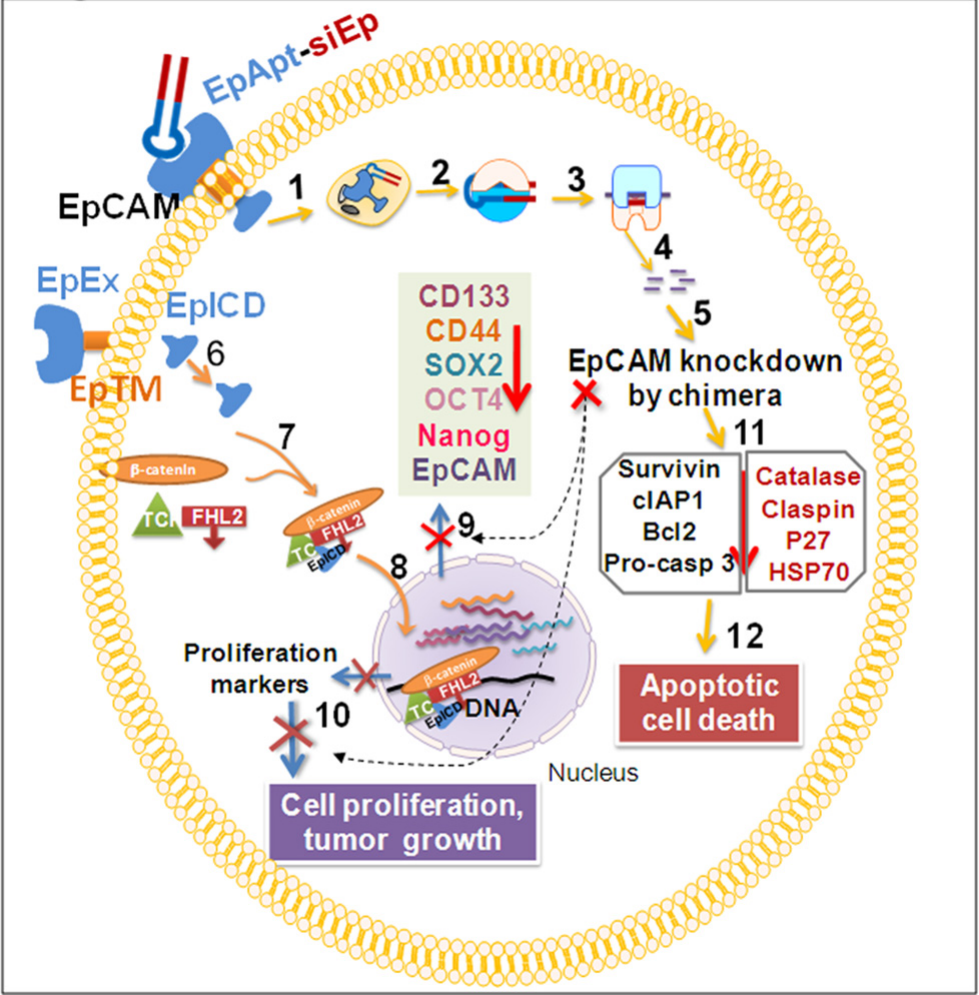
Illustration summarizing the EpCAM aptamer siRNA chimera effect on the tumor growth inhibition. The EpCAM aptamer siRNA chimeric construct (EpApt-siEp) binds to the EpCAM receptor and gets internalized (1) and released in the cytoplasm where gets into Ago complex with dicer enzyme (2) to generate siRNA. The siRNA loaded into RISC complex (3) binds to the EpCAM mRNA (4) and leads to mRNA degradation (5). The EpCAM proteolysis leads to release of EpICD (EpCAM intracellular domain), shedding of EpEx (EpCAM extracellular domain) from EpTM (EpCAM transmembrane domain) (6) and the EpICD complexes with Wnt signaling mediators, β-catenin, FHL2 and TCF (7) to translocate to nucleus and regulate the gene transcription of pluripotency markers, SOX2, OCT4, NANOG, EpCAM, CD133, CD44 and aids in proliferation (8) upon EpCAM silencing these markers are downregulated (9) and cell proliferation is hampered(10). The EpCAM silencing leads to apoptotic cell death by downregulation of pluripotency, CSC markers, survivin, IAPs, Bcl2, p27, HSP70, catalase and claspin, by affecting intrinsic apoptotic pathway (11) leading to cell death(12). Overall, the knockdown of EpCAM using EpApt-siEp chimera leads to the inhibition of the nuclear signaling mediated by the EpICD, thereby decreases the cancer stem cell marker expression and induces apoptosis that brings down the tumorigenicity.

Immune elicitation is commonly observed phenomenon in the case of antibody but not in aptamer based therapies, they showed higher levels of TNFα and interferon-γ in comparison to untreated candidates[47]. Cytokine profiling and analysis in mice treated with EpApt-siEp showed increased levels of G-CSF, IL1RA, CXCL1, sICAM1, where in IL1RA can potentially block the inflammatory process mediated through IL1. Decrease in TIMP1, M-CSF could be the effect of treatment, as these are reported to be elevated in cancerous conditions as well in poor survival of the candidates[48]. No significant changes in the TNFα or INF-γ levels were observed during the treatment, which are desirable. Also the apoptotic protein profiling revealed that the EpApt-siEp adopts the intrinsic pathway to mediate apoptosis. The inhibitor of apoptosis (IAPs) proteins was greatly downregulated with upregulation of Bax and Bad. Also the increase in cleaved Caspase-3, HSP60 and decrease in claspin, HSP70, catalase supports the mechanism of apoptosis onset [49, 50]. PCNA localizes to nucleus in actively dividing cell, while translocate to cytoplasm in quiescent condition. The vehicle control cells showed nuclear positivity in 50% of cells wherein the EpApt-siEp treated cells showed diffused and faint nucleo-cytoplasmic staining dueto decrease in the proliferation of tumor cells. The above described mechanism and pathways by which the EpCAM aptamer siRNA chimera functions are summarized in Fig 7.

In conclusion, we were able to show the functional activity of chimeric construct by targeted delivery to the EpCAM expressing cells and silencing of EpCAM expression. The observation of the EpICD in the RB opens up avenue for targeting EpCAM with Wnt signaling inhibitors which can synergistically enhance the therapeutic activity. The role of EpICD in regulating the cancer stem cell markers in RB was elucidated for the first time and our group has already shown the relevance behind EpCAM and mir-17-92 cluster in RB. Upon treating the MCF7 xenograft model which expresses higher levels of EpCAM, we were able to elucidate for the first time the tumor regression with our novel EpApt-siEp construct. As EpCAM is overexpressed in many cancers, our study also paves a way for the application of the potential anti-tumor agent in future clinical studies for other cancers.

## Supporting Information

S1 Fig Supporting figure showing the *In vitro* dicer cleavage assay, stability of EpApt-siEp chimeric construct and uptake in MCF7. The reactions after terminating by adding stop solution were run on 2% agarose gel. Controls such as EpCAM aptamer and siRNA alone are run alongside. B. EpCAM aptamer siRNA chimeric construct was incubated in media, media with 10% FBS and in FBS alone upto 96hrs. After the 96hr time point reactions were electrophoresed on 2% agarose gel.
(TIF)

S2 Fig EpCAM knockdown using EpApt-siEp construct in RB primary tumor cells. **A**. Microscopic images showing the expression of GFP transfected with lipofectamine 2000 in RB primary cells. 24hr after transfection cells were imaged with 20X objective. Cellular changes accompanying knockdown of EpCAM knockdown using EpApt-siEp construct in primary RB cells, WERI-Rb1 and MCF7. **B.** The EpCAM mRNA levels were quantified by SYBR green based qPCR from the cDNA of control, siEp and EpApt-siEp treated RB primary tumor cells. The graph shows the EpCAM mRNA levels normalized to β-2-microglobulin as housekeeping gene. **C**. The cellular cytotoxicity analysis of the RB cells with treatments was performed by calculating the LDH activity and normalization with untreated control cells.
(TIF)

S3 Fig Effect of EpApt-siEp on the growth, biochemical parameter and histopathology of MCF7 xenografts. Graph showing the (**A**) Mean body weight change(**B**) % differential leukocyte count (**C**) and biochemical parameters, Blood urea nitrogen (BUN), creatinine, SGPT (serum glutamic pyruvic transaminase) and SGOT (Serum glutamic oxaloacetic transaminase) (on its right) of the Vehicle control group injected with PBS subcutaneously near the tumor site, EpApt-siEp subcutaneously injected near the tumor site on alternate days. **D**. H & E staining of xenograft tumor sections of vehicle control and EpApt-siEp (RNA oligo labeled) was performed after 33days of treatment. The Photographs are taken at 40X magnification. H & E staining of tumor, kidney, lung, spleen, heart and liver section of vehicle control and EpApt-siEp (also labeled as RNA oligo). Mitotic Fig. (White arrow); Fibro-vascular stroma (Yellow arrow); Apoptotic Fig. (Red arrow); Neutrophil (Green arrow); PT- portal triad; CV- central vein; Hp- hepatocytes; A-Alveoli; BV- Blood vessel; WP- White Pulp; RP- Red pulp; T- Tubules; G- Glomeruli.
(TIF)

S1 File Supporting information showing the detailed methods and references for the supplementary data. (DOCX)

S2 File Supporting information file showing the unedited images. Images of unedited blots of Fig 2 and mice, excised tumors of Fig 4.
(PDF)

S1 Table Supporting information showing the list of aptamer-siRNA chimeras used for targeted cancer therapy. (XLSX)

S2 Table Supporting information showing the list of primer and aptamer sequences used in the study. (XLSX)

S3 Table Supporting information showing the treatment schedule with EpApt-siEp in epithelial cancer model, MCF7 xenograft. (XLSX)
