# Supplementary figures and images for: EpCAM Aptamer-siRNA Chimera Targets and Regress Epithelial Cancer
Source: PLoS One. 2015 Jul 15;10(7):e0132407. doi: 10.1371/journal.pone.0132407 (PMC4503753; doi:10.1371/journal.pone.0132407)

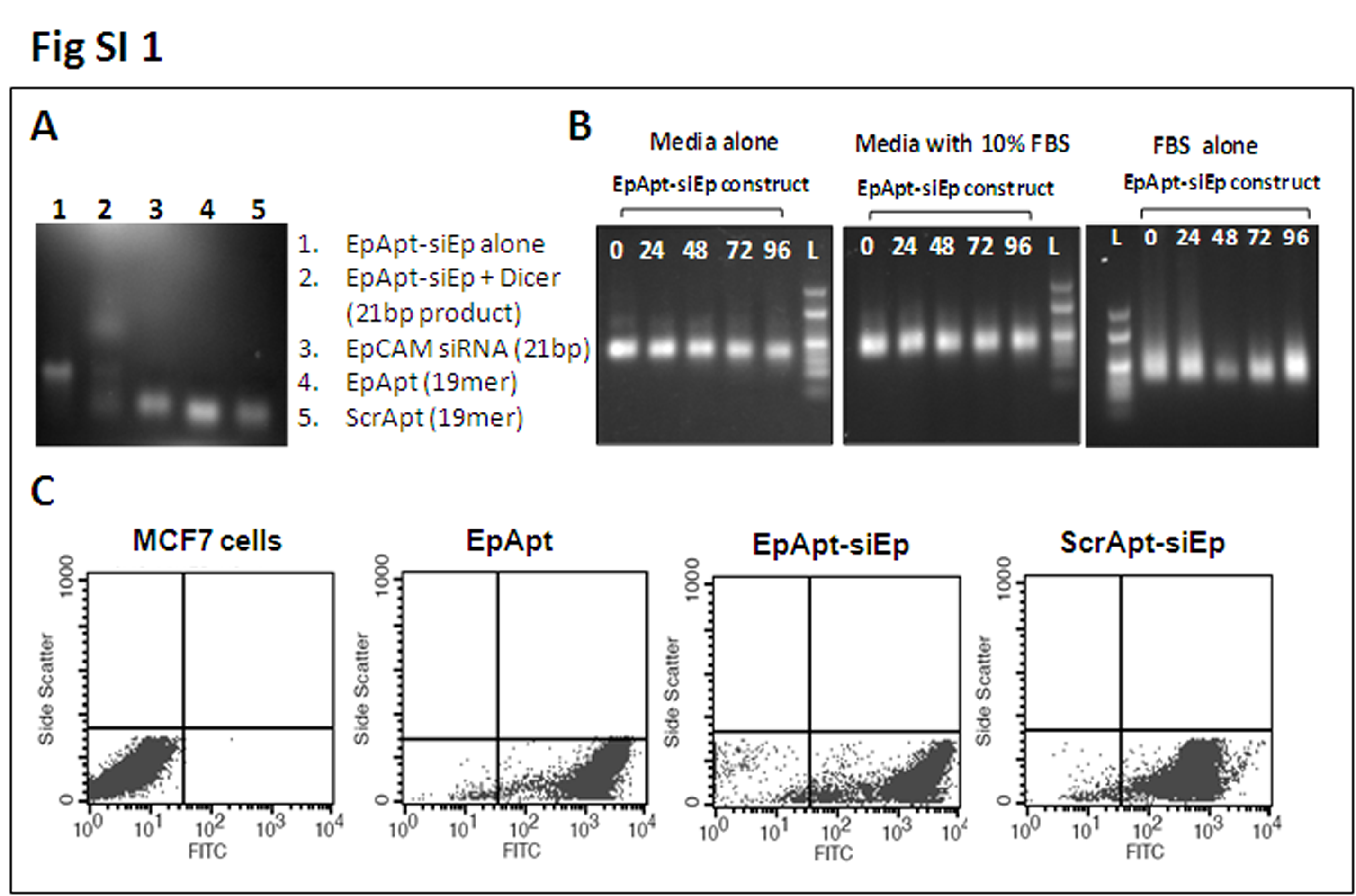

Supplement: S1 Fig — The reactions after terminating by adding stop solution were run on 2% agarose gel. Controls such as EpCAM aptamer and siRNA alone are run alongside. B. EpCAM aptamer siRNA chimeric construct was incubated in media, media with 10% FBS and in FBS alone upto 96hrs. After the 96hr time point reactions were electrophoresed on 2% agarose gel. (TIF) [file pone.0132407.s001.tif]

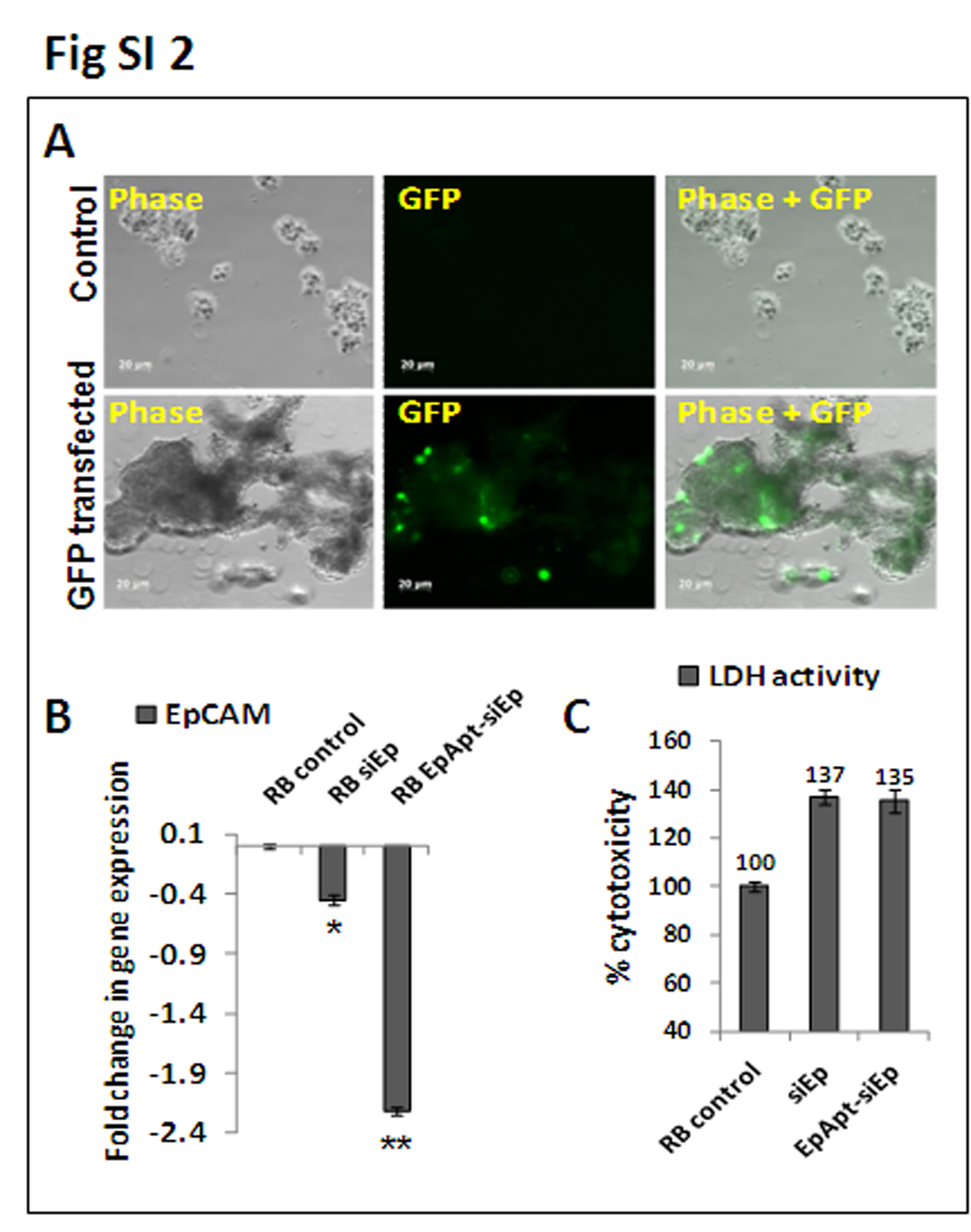

Supplement: S2 Fig — A. Microscopic images showing the expression of GFP transfected with lipofectamine 2000 in RB primary cells. 24hr after transfection cells were imaged with 20X objective. Cellular changes accompanying knockdown of EpCAM knockdown using EpApt-siEp construct in primary RB cells, WERI-Rb1 and MCF7. B. The EpCAM mRNA levels were quantified by SYBR green based qPCR from the cDNA of control, siEp and EpApt-siEp treated RB primary tumor cells. The graph shows the EpCAM mRNA levels normalized to β-2-microglobulin as housekeeping gene. C. The cellular cytotoxicity analysis of the RB cells with treatments was performed by calculating the LDH activity and normalization with untreated control cells. (TIF) [file pone.0132407.s002.tif]

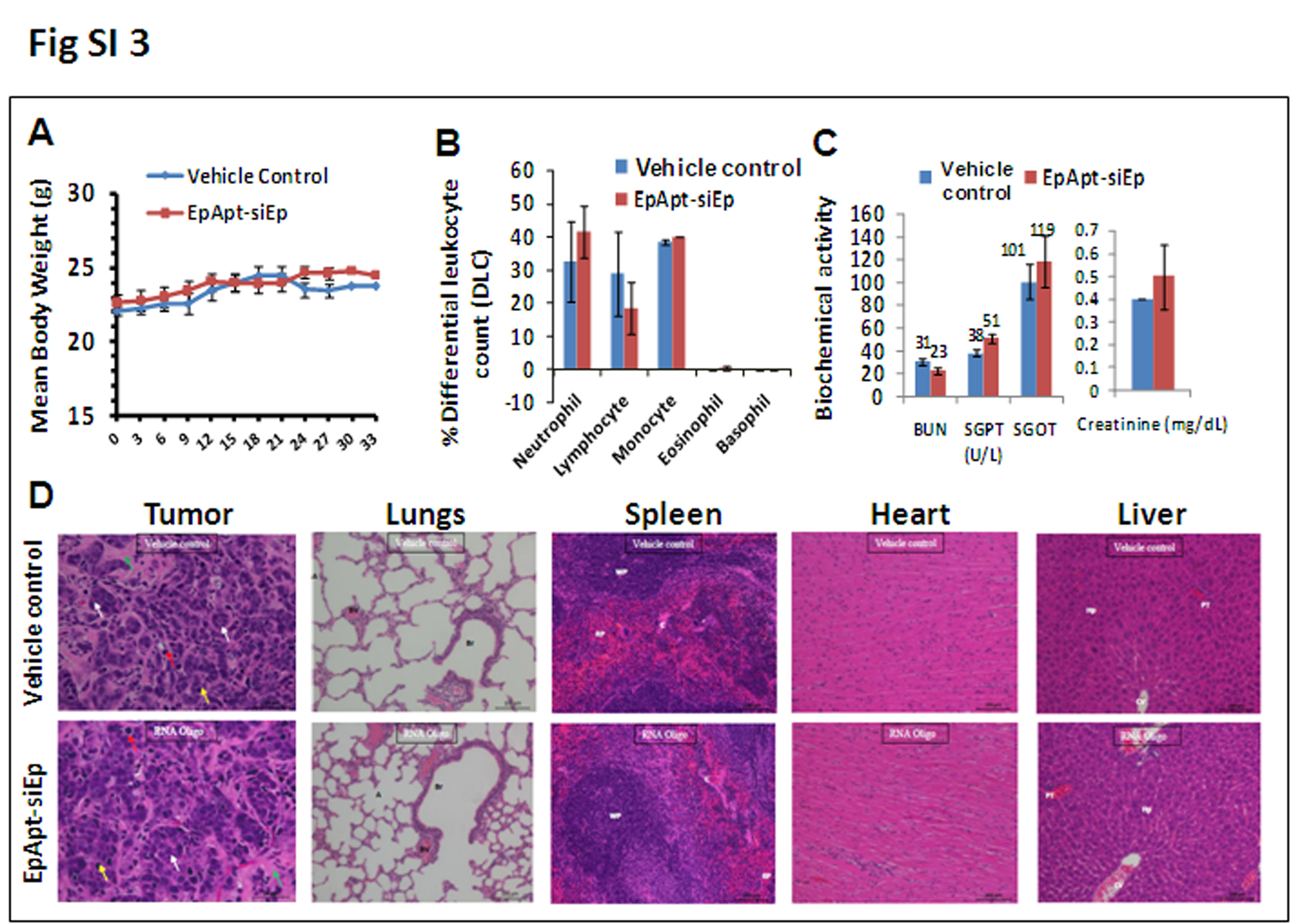

Supplement: S3 Fig — Graph showing the (A) Mean body weight change(B) % differential leukocyte count (C) and biochemical parameters, Blood urea nitrogen (BUN), creatinine, SGPT (serum glutamic pyruvic transaminase) and SGOT (Serum glutamic oxaloacetic transaminase) (on its right) of the Vehicle control group injected with PBS subcutaneously near the tumor site, EpApt-siEp subcutaneously injected near the tumor site on alternate days. D. H & E staining of xenograft tumor sections of vehicle control and EpApt-siEp (RNA oligo labeled) was performed after 33days of treatment. The Photographs are taken at 40X magnification. H & E staining of tumor, kidney, lung, spleen, heart and liver section of vehicle control and EpApt-siEp (also labeled as RNA oligo). Mitotic Fig. (White arrow); Fibro-vascular stroma (Yellow arrow); Apoptotic Fig. (Red arrow); Neutrophil (Green arrow); PT- portal triad; CV- central vein; Hp- hepatocytes; A-Alveoli; BV- Blood vessel; WP- White Pulp; RP- Red pulp; T- Tubules; G- Glomeruli. (TIF) [file pone.0132407.s003.tif]
