# Supplementary material for: EpCAM Aptamer-siRNA Chimera Targets and Regress Epithelial Cancer
Source: PLoS One. 2015 Jul 15;10(7):e0132407. doi: 10.1371/journal.pone.0132407 (PMC4503753; doi:10.1371/journal.pone.0132407)
Supplement: S1 File — (DOCX) [file pone.0132407.s004.docx]

**Supplementary methods:**

***In vitro* dicer cleavage assay:**

*In vitro* dicer Assay was performed to show that our EpApt-siEp chimera is being processed by dicer for the release of siRNA from the aptamer. To ensure proper secondary structure formation of the RNA chimeric construct, 1 µg of the sample suspended in a total volume of 10 µl nuclease free water was heated in boiling water (to denature the sample) for 10 min and allowed to gradually cool (to anneal the sample) to RT. A 10 µl reaction was set up by using 500ng of the annealed RNA chimera, 1 µl of 10 mM ATP, 0.5 µl of 50 mM Mgcl2, 4 µl of 10X Dicer buffer and 2µl (1 Unit) of Dicer enzyme. A control reaction was also set up without the enzyme. Both the reactions are incubated at 37°C for 18h and 2µl stop solution was added to stop the enzyme activity. The samples were run on a 15% Polyacrylamide Gel and visualized on a UV transilluminator (Recombinant Human Dicer Enzyme Kit Cat No: T510002).

**Aptamer uptake study:**

Cellular uptake of FITC labeled aptamer chimeras by the target cells was studied by flow cytometry and fluorescent microscopy. 2X10^5^ MCF7 and WERI-Rb1 cells were incubated with 50, 250, 500nM aptamer-siRNA chimera for 2 hours in RPMI complete media. After incubation, the cells were collected, washed twice with 1X PBS and acquired by flow cytometer. A 24 well plate containing coverslips that are coated with poly-l-lysine were seeded with 50,000 cells and allowed to grow for 24 hours. The cells were washed twice with media and were treated with 400nM Aptamer-siRNA chimera for 2 hours. The Scrambled chimera was used as a control. The cells were washed twice with 1X PBS, fixed in 3.7% formaldehyde for 10 min, stained with DAPI (1:50000) (Sigma Aldrich, India) for 15 min. The coverslips with cells were washed with 1X PBS and observed under Axio-Observer fluorescent microscope.

**RNA isolation and quantitative real time PCR:**

Total RNA was isolated by Tri-reagent method. The quality and quantity of the RNA was assessed by Biospec-Nano spectrophotometer and 1 µg of RNA was reverse transcribed to cDNA using a verso kit. qPCR was performed by SYBR green method using Dynamo HS mastermix for EpCAM, β-2-mocroglobulin, SOX2, OCT4 and NANOG. The reaction conditions were initial denaturation at 95°C for 10 min followed by 40 cycles of denaturation at 95°C for 30 sec, annealing at 55°C for 45 sec, extension at 72°C for 45 sec. Final extension was for 10 min at 72°C. Meltcurve analysis was performed to verify the specificity of amplification. Comparative quantification was performed using the formula 2^-ddct^ and the log2 values are expressed as fold change regulation. The list of primer and aptamer sequence used is given in supplementary table 2.

**Northern blotting:**

Total RNA was isolated from MCF7 untreated cells, siRNA transfected cells and Aptamer-siRNA chimera treated cells by tri-reagent method. 2X RNA sample buffer (200 µl) was prepared by adding 100 µl of redistilled formamide, 33 µl of 37% formaldehyde solution, 4 µl of 0.5M EDTA (pH 8), 8 µl of 1M NAP (pH 6.8), 4 µl of EtBr (10 mg/ml) and 51 µl of DEPC treated water. The RNA samples (3 µg each) were added to equal volume of 2X RNA sample buffer and was heated at 65°C for 15 min to remove secondary structures. The samples were electrophoresed at 100V in 1% denaturing NAP-formaldehyde agarose gel and NAP-formaldehyde running buffer made with DEPC treated water. The electrophoresed samples were transferred onto a nylon membrane by capillary transfer and cross-linked by baking at 80°C for 15 min. EpCAM siRNA was labeled with Biotin by Brightstar Psoralen-Biotin Kit method (Cat No: AM1480) and was used as a probe. The probe (100 ng) was hybridized onto the membrane by using ULTRAhyb-Oligo buffer (Cat No: AM8663) and EpCAM mRNA level was detected by using BrightStar BioDetect Kit (AM1930) by autoradiography.

**Western blotting:**

MCF7 and WERI-Rb1 cells were cultured and treated in 6 well plates and collected by centrifugation. To the cell pellet ten bed volumes of 1X RIPA buffer was added, incubated at 4˚C for 1h followed by centrifugation at high speed 10min to yield cell lysate. The lysate was run on 12% SDS-PAGE using BIORAD’S apparatus. The proteins are transferred onto a nitrocellulose membrane by wet transfer method for an hr and the blot was visualized by Ponceau stain for protein transfer. The blot was washed, blocked with 5% skim milk for an hr at RT, followed by incubation with EpCAM primary antibody (1:1000) (C-10, Santacruz, USA), β-tubulin (1:1000) (Santacruz, USA), anti-mouse ABCG2 antibody (1:2000) (Sigma Aldrich, India) and β-actin (1:3000) (Sigma Aldrich, India) followed by secondary anti-mouse HRP antibody (1:3000) (Santacruz, USA) and developed by chemiluminescence method using SuperSignal WestFemto reagent by autoradiography and chemiluminescent imager (Fluorchem FC3, Protein Simple).

**Immunohistochemistry:**

Paraffin embedded tissue sections were deparaffinized by serial treatments of Xylene and ethanol. Immunohistochemistry (IHC) was performed using Novolink polymer detections system (Leica biosystems), following the instructions given by the manufacturer. The tissues were rehydrated and antigen retrieved under steaming in a pressure cooker containing (0.1 M citric acid, 0.1 M trisodium citrate, PH 6) buffer for 4 minutes (two whistles). The cooker was allowed to cool in running water and the tissues were rehydrated and added with 3% H_2_0_2_ for 5 min to block endogenous peroxidase activity. The tissues were washed twice with TBS, treated with protein block solution for 5 min to avoid non specific binding and added with primary antibody, EpICD (IMG6745A,Imgenex,Novusbiologicals) (1:100 dilution) and kept at 4°C overnight. The detection was performed by using Novolink polymer IHC detection kit. The tissues were DAB stained for 5 min, counter stained by hematoxylin for 30 sec, washed twice with water. The slides were dehydrated, mounted with DPX, cover slipped and allowed to cure overnight before observing under Olympus microscope.

**Lactate dehydrogenase (LDH) assay:**

Cell cytotoxicity was studied using cytotoxicity detection kit plus (Cat. No.04744926001, Roche) for lactate dehydrogenase (LDH) activity on untreated control cells vs RB primary cells treated with EpApt-siEp and transfected with siEp using lipofectamine 2000 (Invitrogen) for 48h duration. High control, low controls were used for calculating the percentage cytotoxicity.

**Preparation of tumor cells and subcutaneous injection of cells in Balb/c nude mice**

All procedures were performed in laminar flow hood following sterile techniques. Breast adenocarcinoma cells (MCF7) with a viability of >90 % was chosen for the study. Ideally 5 X 10^6^ MCF7 cells (ATCC) was resuspended in 200 µl of serum free media containing 50% of matrigel kept in ice. Animal study was performed by utilizing the facility of Syngene (Bangalore, India) and experimentations were approved by the Institutional Animal Ethics Committee (IAEC Protocol Approval No: SYNGENE/IAEC/430/10-2013). Animals were maintained in a controlled environment with ambient temperature, humidity and light, fed with irradiated rodent diet, RO filtered potable water and housed group wise. Hsd: Athymic Nude-Foxn1^nu^ mice (female; bilaterally ovariectomized, 7-8 weeks old) was used for the present investigation. The tumorigenicity of the MCF7 cells as a proof of concept for EpCAM positive epithelial cancer cells (estrogen-dependent) were conducted in mice. Twenty hours prior to MCF7 cell injection, animals were implanted with 17β-estradiol pellets (0.36mg/pellet; 60-day release; Innovative Research of America, Sarasota, FL) into dorsal shoulder blade region of mice using trochar. Twenty four hours post implantation of pellets, MCF7 tumor cells (5 x10^6^ cells/animal) were injected subcutaneously in flanks of the animals. The implanted area was monitored for growth of tumor daily. Once the tumor attained palpable and animals were randomized based on tumor volume (TV≈80mm^3^) and dosing was initiated. The treatment schedule is given below in table 3:

The body weights and tumor volume were measured once every three days and % change in body weight was calculated. Tumor volume was determined by two-dimensional measurement with a vernier caliper, the length (L) and width (W) of the tumor was measured. Tumor volume (TV) was calculated using the following formula: Tumor Volume (mm^3^) = L x W^2^ / 2, Where, L = Length; W = Width. Mean and Standard Error of Mean (SEM) were calculated for individual groups and plotted as graph. Tumor growth inhibition was calculated using the formula: TGI = (1 - T/C) x 100, where, T = (Mean TV of the test group on Day _X_ - Mean TV of the test group on Day_0_) and C = (Mean TV of the control group on Day _X_ -Mean TV of the control group on Day_0_).

**Blood cell counts, biochemical and histology analysis**

At the end of the experiment period, on the day of sacrifice, blood was collected under isoflurane anesthesia from all the groups for clinical assessment of liver function (SGOT, SGPT) & kidney function (BUN, Urea). Additionally peripheral blood smears were prepared and were stained with May-Grunwald Giemsa stain and evaluated for differential leukocyte count (DLC). Then the animals were sacrificed and representative animals from each group were photographed (Nude mice bearing tumor and harvested tumor alone). The subcutaneous tumor (MCF7) tissue was harvested and divided into two parts (snap frozen, formalin fixed) for further analysis. Necropsy was done to evaluate gross evidence of possible lesions in organs such as lungs, liver, spleen, kidney and heart. The collected organs were stored in 10% neutral buffered formalin, dehydrated and fixed paraffin blocks followed by sectioning for the histological analysis. Haematoxylin and eosin staining was performed on the organs and tumor section.

**Reference**:

DASSIE, J. P., LIU, X. Y., THOMAS, G. S., WHITAKER, R. M., THIEL, K. W., STOCKDALE, K. R., MEYERHOLZ, D. K., MCCAFFREY, A. P., MCNAMARA, J. O., 2ND & GIANGRANDE, P. H. 2009. Systemic administration of optimized aptamer-siRNA chimeras promotes regression of PSMA-expressing tumors. *Nat Biotechnol,* 27**,** 839-49.

HUSSAIN, A. F., TUR, M. K. & BARTH, S. 2013. An aptamer-siRNA chimera silences the eukaryotic elongation factor 2 gene and induces apoptosis in cancers expressing alphavbeta3 integrin. *Nucleic Acid Ther,* 23**,** 203-12.

LAI, W. Y., WANG, W. Y., CHANG, Y. C., CHANG, C. J., YANG, P. C. & PECK, K. 2014. Synergistic inhibition of lung cancer cell invasion, tumor growth and angiogenesis using aptamer-siRNA chimeras. *Biomaterials,* 35**,** 2905-14.

MCNAMARA, J. O., 2ND, ANDRECHEK, E. R., WANG, Y., VILES, K. D., REMPEL, R. E., GILBOA, E., SULLENGER, B. A. & GIANGRANDE, P. H. 2006. Cell type-specific delivery of siRNAs with aptamer-siRNA chimeras. *Nat Biotechnol,* 24**,** 1005-15.

NI, X., ZHANG, Y., RIBAS, J., CHOWDHURY, W. H., CASTANARES, M., ZHANG, Z., LAIHO, M., DEWEESE, T. L. & LUPOLD, S. E. 2011. Prostate-targeted radiosensitization via aptamer-shRNA chimeras in human tumor xenografts. *J Clin Invest,* 121**,** 2383-90.

SHAW, B. R., MOUSSA, L., SHARAF, M., CHEEK, M. & DOBRIKOV, M. 2008. Boranophosphate siRNA-aptamer chimeras for tumor-specific downregulation of cancer receptors and modulators. *Nucleic Acids Symp Ser (Oxf)***,** 655-6.

THIEL, K. W., HERNANDEZ, L. I., DASSIE, J. P., THIEL, W. H., LIU, X., STOCKDALE, K. R., ROTHMAN, A. M., HERNANDEZ, F. J., MCNAMARA, J. O., 2ND & GIANGRANDE, P. H. 2012. Delivery of chemo-sensitizing siRNAs to HER2+-breast cancer cells using RNA aptamers. *Nucleic Acids Res,* 40**,** 6319-37.

WULLNER, U., NEEF, I., ELLER, A., KLEINES, M., TUR, M. K. & BARTH, S. 2008. Cell-specific induction of apoptosis by rationally designed bivalent aptamer-siRNA transcripts silencing eukaryotic elongation factor 2. *Curr Cancer Drug Targets,* 8**,** 554-65.

ZHOU, J., TIEMANN, K., CHOMCHAN, P., ALLUIN, J., SWIDERSKI, P., BURNETT, J., ZHANG, X., FORMAN, S., CHEN, R. & ROSSI, J. 2013. Dual functional BAFF receptor aptamers inhibit ligand-induced proliferation and deliver siRNAs to NHL cells. *Nucleic Acids Res,* 41**,** 4266-83.
