# Supplementary material for: EpCAM Aptamer-siRNA Chimera Targets and Regress Epithelial Cancer
Source: PLoS One. 2015 Jul 15;10(7):e0132407. doi: 10.1371/journal.pone.0132407 (PMC4503753; doi:10.1371/journal.pone.0132407)

Fig 2A supporting image

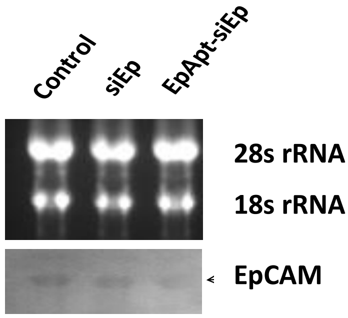

EpCAM antisense oligo probed and developed by autoradiography

MCF7 total RNA samples run on formaldehyde agarose gel

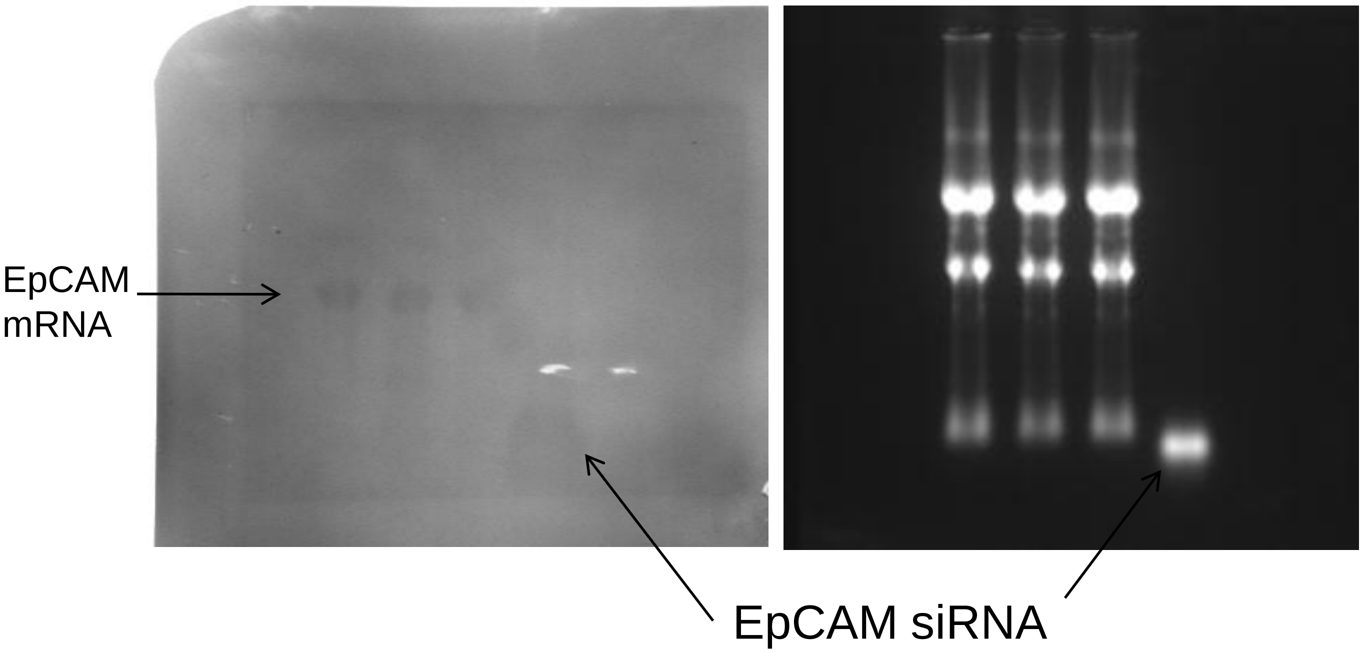

Fig 2C supporting image

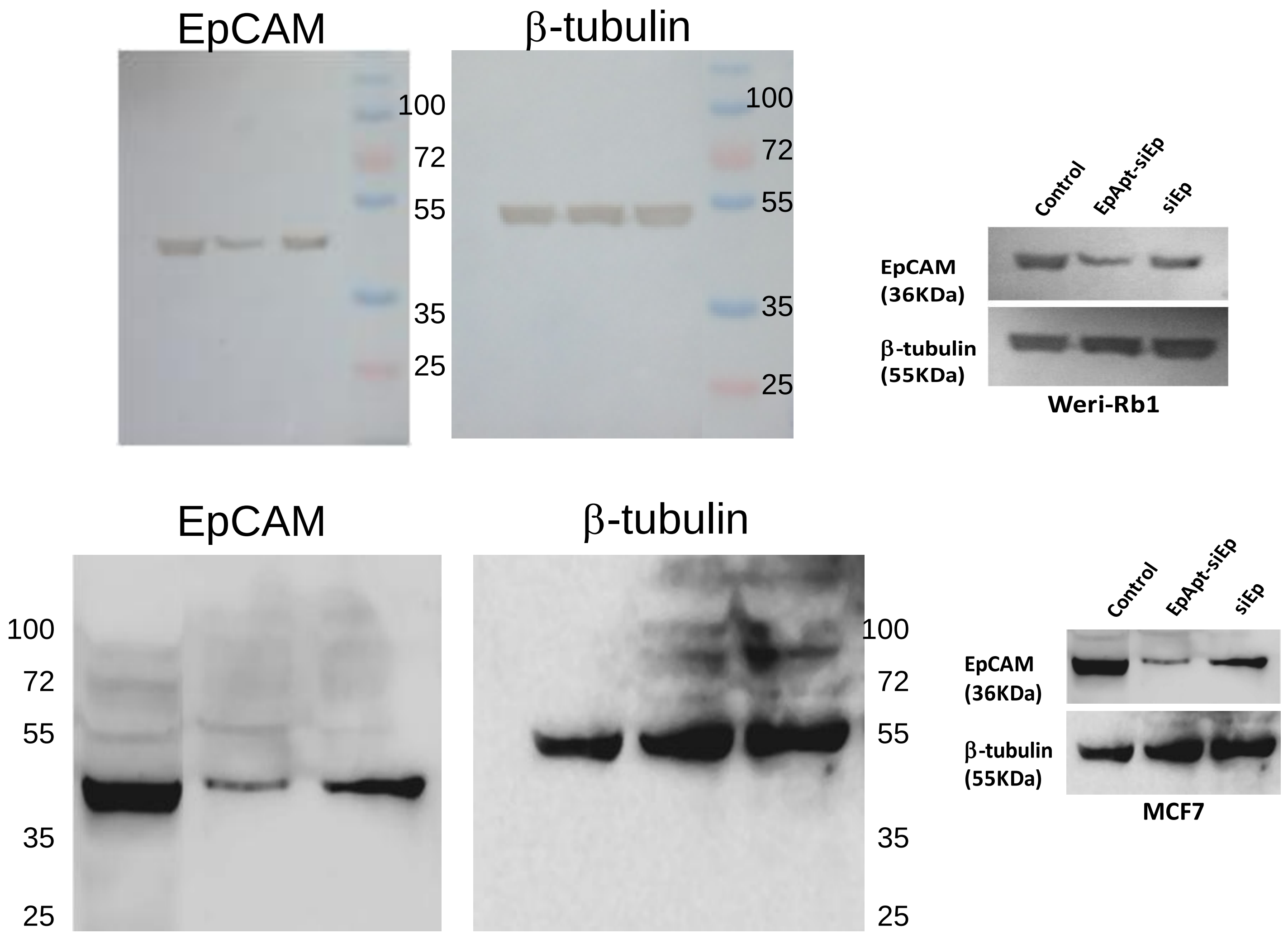

Fig 4D supporting image

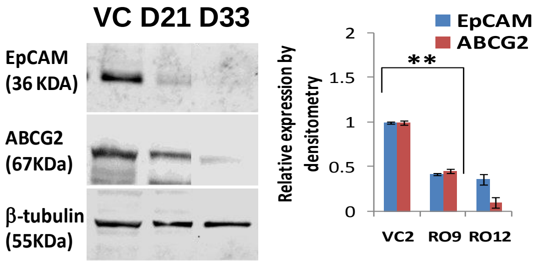

Immunoblot assembled showing the results used in blue box
(samples in graph showing the actual label in the uncropped image)

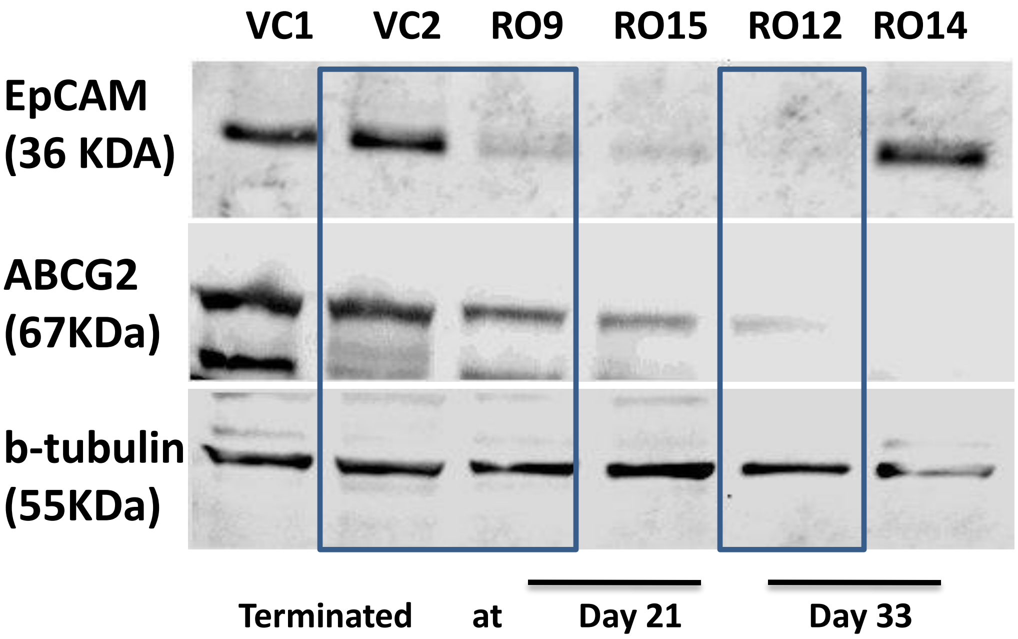

Fig 4B & C supporting image

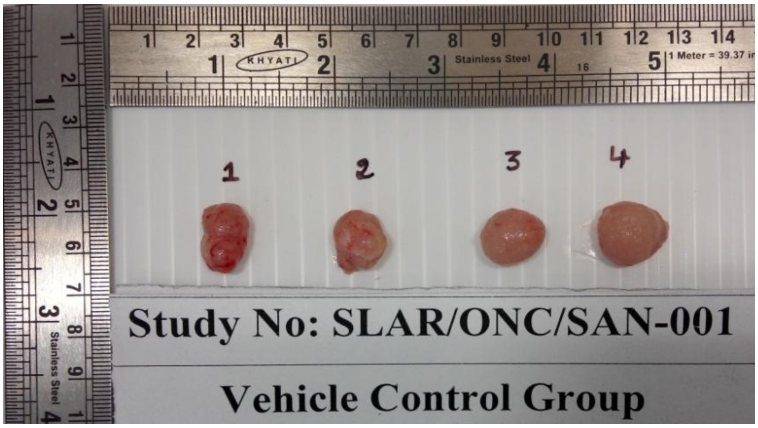

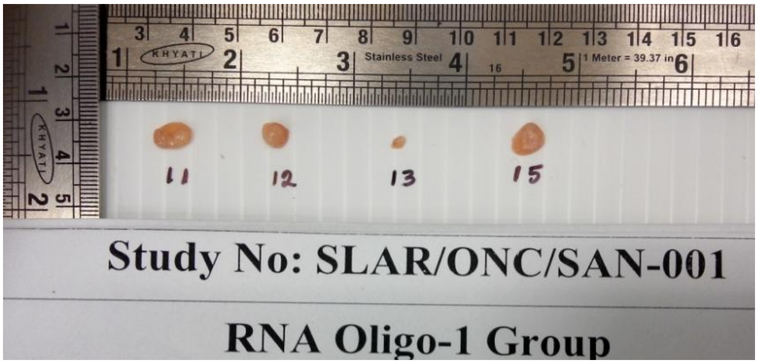

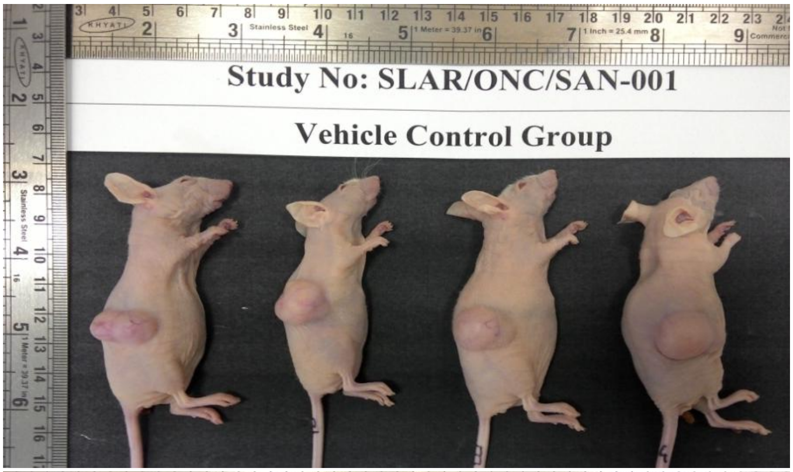

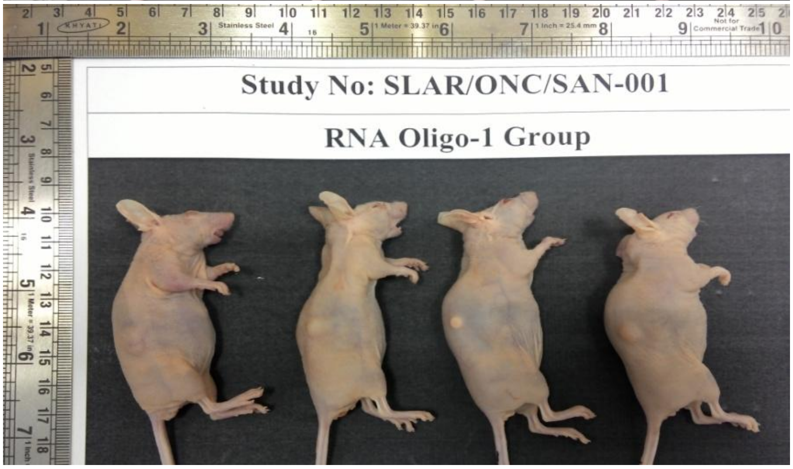

Supplement: S2 File — Images of unedited blots of Fig 2 and mice, excised tumors of Fig 4. (PDF) [file pone.0132407.s005.pdf]
